# Ancestral Sequence Reconstruction of the Ethylene-Forming Enzyme

**DOI:** 10.1021/acs.biochem.5c00334

**Published:** 2025-07-25

**Authors:** Shramana Chatterjee, Joel A. Rankin, Mark A. Farrugia, Bryce J. Delaney, Nathaniel S. Pascual, James VanAntwerp, Daniel R. Woldring, Jian Hu, Robert P. Hausinger

**Affiliations:** 1 Department of Microbiology, Genetics, and Immunology, 3078 Michigan State University, East Lansing, Michigan 48824, United States; 2 Department of Chemical Engineering and Materials Science, 3078 Michigan State University, East Lansing, Michigan 48824, United States; 3 Institute for Quantitative Health Science and Engineering, 3078 Michigan State University, East Lansing, Michigan 48824, United States; 4 Department of Biochemistry and Molecular Biology, 3078 Michigan State University, East Lansing, Michigan 48824, United States; 5 Department of Chemistry, 3078 Michigan State University, East Lansing, Michigan 48824, United States

## Abstract

The ethylene-forming enzyme (EFE) catalyzes two main reactions: the conversion of 2-oxoglutarate (2OG) to ethylene plus CO_2_ and the oxidative decarboxylation of 2OG coupled to the C5 hydroxylation of l-arginine (l-Arg). EFE also facilitates two minor reactions: the uncoupled oxidative decarboxylation of 2OG and the generation of 3-hydroxypropionate (3HP) from 2OG. To better understand the evolution of this enzyme’s diverse activities, we demonstrated that two distantly related extant enzymes produce trace levels of ethylene and 3HP, and we examined the reactivities of 11 reconstructed ancestors. The structure of one ancestral protein was resolved by X-ray crystallography, while the others were modeled with AlphaFold2. These studies highlight the importance of residues located at the 2OG and l-Arg binding pockets for the varied activities. For example, effective formation of ethylene requires that the 2OG binding pocket be hydrophobic except for interactions with the substrate carboxylates. Newly identified changes near the l-Arg binding site exhibit significant effects on the reactivities of the enzyme's reactions. Analysis of the reconstructed ancestors suggests that the primordial enzyme exhibited both ethylene-forming and l-Arg hydroxylation activities with partition ratios like the extant examples; i.e., an enzyme capable of catalyzing predominantly one of these reactions did not subsequently develop the ability to affect the secondary reaction.

## Graphical abstract

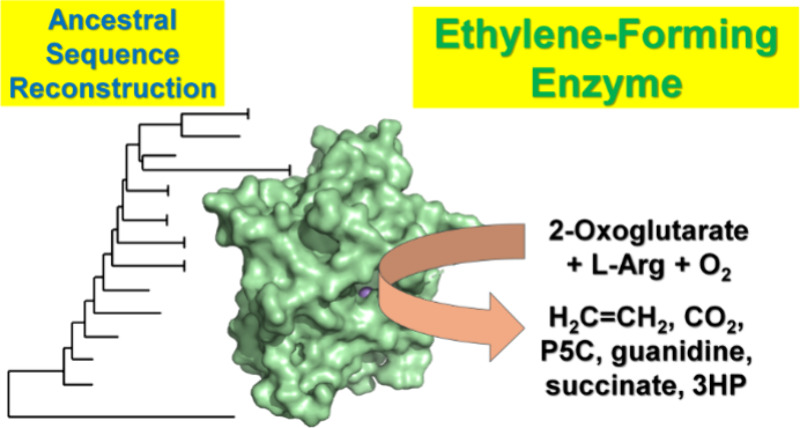

## Introduction

Ethylene is a key industrial chemical used in the production of transportation fuel, plastics, and other important organic compounds;^1,2^ however, its current commercial synthesis via fossil fuel steam cracking is the most CO_2_-intensive process in the chemical industry.^3,4^ Consequently, there is significant interest in developing more sustainable production methods using renewable sources.^5–7^ One approach to address this need is to utilize naturally occurring biological sources of ethylene,^8^ especially that associated with the microbial ethylene-forming enzyme (EFE). EFE utilizes 2-oxoglutarate (2OG) and l-arginine (l-Arg) as substrates and catalyzes multiple reactions, most notably the unique l-Arg-dependent conversion of 2OG to ethylene and CO_2_ (Figure 1A).^9^ In addition to this transformation, EFE catalyzes the oxidative decarboxylation of 2OG coupled to the C5 hydroxylation of l-Arg, producing an intermediate that spontaneously decays to guanidine and l-Δ^1^-pyrroline-5-carboxylate (P5C) **(** Figure 1B).^10^ As observed for other Fe­(II)/2OG-dependent oxygenases,^11,12^ the uncoupled decarboxylation of 2OG also occurs in the presence of a reductant (Figure 1C).^13^ Finally, when provided with 2OG and l-Arg, EFE was computationally and experimentally shown to produce 3-hydroxypropionate (3HP) (Figure 1D),^14–17^ a biodegradable plastic precursor.^18^ While previous biochemical and structural studies have primarily focused on EFE from *Pseudomonas savastanoi* (formerly *P. syringae*) strain PK2 (PK2 EFE),^10,14,15,19–22^ recent analyses of a fungal EFE from *Penicillium digitatum* strain Pd1 (Pd1 EFE) revealed a higher ethylene-to-P5C partition ratio,^13^ suggesting enhanced ethylene-forming efficiency and highlighting the promise of harnessing the diversity of microbial EFEs to advance sustainable ethylene production.

**Figure 1:**
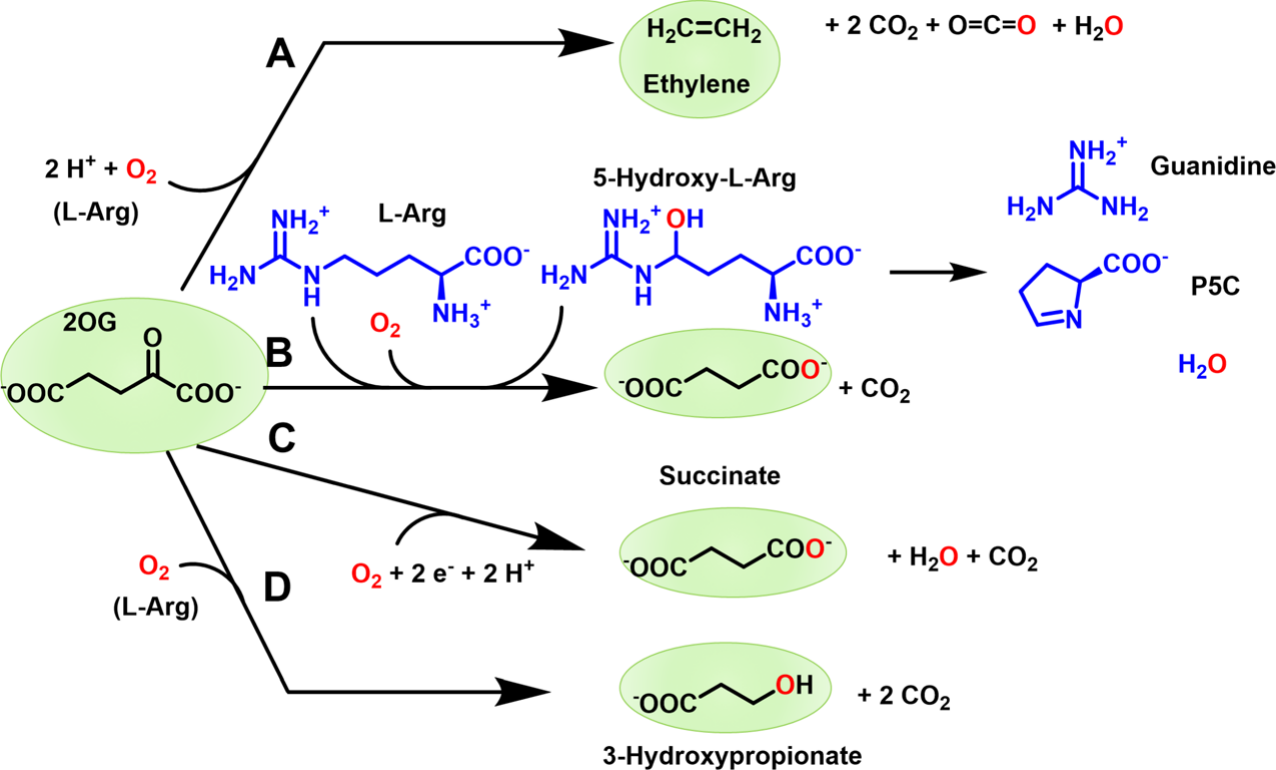
Reactions catalyzed by EFE. (A) The major reaction generates ethylene from 2OG in the presence of l-Arg. (B) Oxidative decarboxylation of 2OG drives l-Arg hydroxylation with subsequent spontaneous degradation to guanidine and P5C. (C) Uncoupled oxidative decarboxylation. (D) 3HP production from 2OG in the presence of l-Arg.

The evolutionary origin of EFE remains an open question, particularly given its ability to catalyze multiple distinct reactions using a common set of cofactors and substrates. One possibility is that an ancestral EFE enzyme evolved in the context of amino acid metabolism, catalyzing the hydroxylation or oxidative degradation of l-Arg as a primary function. During this process, trace levels of ethylene might have been produced as a nonessential byproduct. Over time, the trace production of this plant hormone may have conferred a selective advantage to a plant pathogen, providing evolutionary pressure for the development of a more efficient ethylene-forming activity as observed in present-day EFEs. The presence of annotated EFE homologues in diverse bacterial and fungal species suggests an ancient origin. Analysis of structurally or mechanistically similar extant enzymes that are distant in sequence also can provide insights into the evolutionary trajectory of EFE. In addition, ancestral sequence reconstruction (ASR, the detailed approaches are described in Methods),^23^ a computational technique which infers the sequences of ancient proteins based on the evolutionary relationships of modern homologues, could provide insights into the function of the primordial enzyme. In this study, we combined experimental and computational approaches to better understand the ancestry of EFE. Specifically, we experimentally characterized the biochemical and biophysical properties of resurrected proteins to reveal how their structure and function evolved over time. This information may then allow one to introduce further protein engineering changes for the generation of desired products.^24–26^

Here, we attempt to learn more about EFE ancestry by following three lines of inquiry. First, we examined a member of the isopenicillin N synthase (IPNS) oxygenase family from *Pseudomonas aeruginosa* PAO1 (*Pa*IPNS)^27^ that is structurally similar to EFE [with a root-mean-square deviation (RMSD) of 1.3 Å for the Cα atoms when comparing chain A of PK2 EFE·Ni­(II) (protein data bank, PDB: 5V2V) and chain A of *Pa*IPNS·Na (PDB: 6JYV)]. Whereas IPNS transforms l-δ-(α-aminoadipoyl)-l-cysteinyl-d-valine (ACV) into isopenicillin N, *Pa*IPNS does not bind ACV and has no known function.^27^ We tested whether *Pa*IPNS is capable of catalyzing any of the EFE catalytic reactions. Second, we investigated the Din11 homologue of homoarginine 6-hydroxylase (HA6H) from *Arabidopsis thaliana* (*At*HA6H) that also catalyzes C5 hydroxylation of l-Arg. Given its catalytic similarity to EFE, we examined whether the Din11 *At*HA6H exhibits other EFE-like reaction capabilities. Finally, we use ASR methods^23^ to identify potential precursors of EFE, then synthesize and characterize the properties of those enzymes including the crystal structure for one case. We find that the ancestors possess varying levels of ethylene-forming and l-Arg hydroxylation activities with a near uniform partition ratio, inconsistent with the hypothesis that extant versions of EFE evolved from an enzyme with a single activity. Many of the reconstructed ancestral proteins exhibit significant activity for the uncoupled oxidative decarboxylation of 2OG and we detected widespread 3HP production, allowing us to speculate on alternative functional roles of the precursor enzyme.

## Methods

### Phylogenetic Analysis and Ancestral Sequences Reconstruction

The sequence for *P. digitatum* strain Pd1 EFE (Uniprot A0A7T7BQH3) was queried by BLAST using the nonredundant protein sequence databank.^28^ The 1000 homologues obtained were filtered to remove highly similar sequences (>90% identical) and duplicates by CD-HIT.^29^ The remaining 344 sequences were aligned using the MUSCLE algorithm.^30^ A Whelan and Goldman substitution model with gamma distributed invariant sites was used to generate the phylogenetic tree using MEGA X software.^31^ Ancestral sequences for all proteins were constructed from the extant proteins of the phylogenetic tree using the maximum likelihood method utilized by MEGA X.

Consecutive analyses were required to identify ancestral proteins with sufficient sequence differences from the initial query protein of *P. savastanoi* strain PK2 EFE (Uniprot P32021). The same process used for Pd1 EFE was repeated on PK2 EFE, with two distantly related IPNS family oxygenases from *Deltaproteobacteria bacterium* (GenBank accession: TMB71635, TMB) and *Phenylobacterium* sp. (GenBank accession THD58955, THD) being identified and utilized in a subsequent BLAST search for 500 homologues of each. CD-HIT cleanup for homologues to TMB and THD resulted in 140 and 149 sequences, respectively, which were used to generate phylogenetic trees using a Whelan and Goldman substitution model with frequencies and gamma distributed invariant sites. Ancestors of interest were identified based on alterations to the active site of EFE, with ancestor 124 (Anc124) being identified from the Pd1 EFE search, Anc357 from the TMB search, and Anc317 from the THD search.

As an independent approach for identifying ancestral sequences, we extracted the evolutionary information for EFE (using the PK2 sequence as input) with AP-LASR,^32^ a software tool that fully automates ASR. This process similarly involved identification of homologues (BLASTp), multiple sequence alignment (MAFFT),^33^ and phylogenetic tree prediction and ASR (IQ-TREE),^34^ with the final alignment containing 399 sequences.^34^ Based on AP-LASR results, we sampled sequences from six high-stability ancestral nodes spanning different evolutionary time scales: Node 10, Node 13, Node 253, Node 326, Node 384, and Node 385 (Figure S1, **left**, with confidence values shown in Table S1). Additionally, we used *Pa*IPNS (UniProt ID: Q9HWJ0) as an outgroup and applied the same reconstruction method. This entailed modifying the original final alignment produced by AP-LASR to include the sequence for *Pa*IPNS as well as additional sequences to fill in the gap between *Pa*IPNS and the sequences in the original final alignment (using NCBI’s BlastP). Like the previous ASR run, these sequences were aligned and clustered with MAFFT and CD-HIT to remove redundant sequences. The final alignment for the *Pa*IPNS outgroup analysis yielded 209 sequences, which enabled us to identify two key ancestral nodes, Node 3 and Node 5 (Figure S1, **right** and Table S1).

### Cloning, Gene Expression, and Purification of Ancestral EFEs and Related Enzymes

The genes corresponding to *Pa*IPNS, Anc124, Anc317, Anc357, Node 10, Node 13, Node 253, Node 326, Node 384, and Node 385 were synthesized with codon optimization by IDT (Coralville, IA) and that for Din11 was synthesized with codon optimization by Twist Bioscience (San Francisco, CA). The genes were incorporated into pET28a containing the T7 expression system, the kanamycin resistance gene, and a sequence encoding an N-terminal His_6_ tag upstream from the inserted genes. An alignment of the protein sequences is shown in Figure S2, and the primer sequences and cloning methods that were used are described in Table S2.

All buffers were prepared at room temperature with the pH adjusted using either NaOH or HCl. Terrific broth medium supplemented with 50 μg/mL kanamycin (1 L in 2.8 L Fernbach flasks) was inoculated (1 or 2%) with overnight cultures of cells containing plasmids for production of *Pa*IPNS, Din11, and the ancestral sequences, then grown at 37 °C with shaking at 200 rpm until reaching an OD_600_ of 0.8–1.2. The temperature of the cultures was lowered to 20 °C, the cells were induced with IPTG (final concentration of 0.2 mM), and growth continued overnight with shaking at 180 rpm. The next day, the cultures were harvested by centrifugation at 7,000 *g* and 4 °C for 15–20 min. The cells were stored at –80 °C until further use.

The cell pellets were thawed, resuspended in buffer A [50 mM NaH_2_PO_4_ (pH 8.0), 500 mM NaCl, and 10 mM imidazole], and supplemented with 1 mM phenylmethylsulfonyl fluoride (from a 100 mM stock in ethanol) and 1 U/mL Benzonase (EMD Millipore). The cell suspensions were lysed by using a French pressure cell at 16,000 psi at 4 °C or by sonication on ice using a Branson Sonifier 450 (15 s on/30 s off cycles at 30% amplitude for a total process time of 10–15 min). Lysates were clarified by centrifugation (45 min at 100,000 *g*) at 4 °C. The clarified lysates were applied to a Ni-loaded nitrilotriacetic acid (NTA) agarose column, unbound proteins were eluted with 10 column volumes of buffer A, and the proteins of interest were eluted with ∼ 5–10 column volumes of buffer B (buffer A with 300 mM imidazole).

The Ni-NTA fractions containing the desired proteins were concentrated, and the buffer was exchanged for 50 mM NaH_2_PO_4_ (pH 8.0) containing 300 mM NaCl and 10 mM imidazole by using a 10 kDa molecular weight cutoff Amicon Ultra-15 centrifugal filter unit (EMD Millipore). The His_6_ tag was removed from all EFE-related proteins by incubation with His_7_-TEV238Δ protease^35^ for 16–18 h at 4 °C, and the EFE/TEV protease mixtures were applied to Ni-NTA columns that had been equilibrated with buffer A. The flow-through fractions and ∼ 7–10 column volumes of buffer A wash were collected, concentrated to 2.5 mL, and buffer exchanged into 25 mM 4-(2-hydroxyethyl)-1-piperazineethanesulfonic acid (HEPES) buffer (pH 8.0) containing 1 mM EDTA and 1 mM dithiothreitol (DTT) using a PD-10 column (Cytiva). Prior to performing any assays, EDTA was removed from the proteins of interest by using a PD-10 column. For long-term storage, the eluted fractions were concentrated, glycerol was added to a final concentration of 5–10%, the samples were flash-frozen in liquid nitrogen, and the enzymes were placed at –80 °C until further use. For comparative studies, we purified the strain PK2 EFE using a previously described protocol.^19^ The homogeneity of the EFE-related samples was assessed by sodium dodecyl sulfate-polyacrylamide gel electrophoresis (SDS-PAGE).

### Metal Analysis

EFE samples (25–150 μL) were mixed with 100 μL of 70% (v/v) nitric acid and digested for 1 h at 100 °C, diluted to 5 mL with water, and examined using an Agilent 8900 Triple Quadrupole inductively coupled plasma mass spectrometer (ICP-MS) at the MSU Quantitative Bio Element Analysis and Mapping (QBEAM) Center to determine the metal contents.

### Anaerobic UV–Visible Spectroscopy

Stock solutions (100 mM) of 2OG and l-Arg were prepared in 25 mM HEPES buffer (pH adjusted to 7.5) in serum vials sealed with butyl rubber stoppers and made anaerobic by several rounds of vacuum degassing and flushing with argon using a vacuum manifold. After degassing, sodium dithionite was added to a final concentration of 2 mM from a 100 mM stock solution. Ferrous ammonium sulfate stock solutions (100 mM) were prepared by several rounds of degassing and flushing with argon inside a sealed serum vial. The Fe­(NH_4_)_2_(SO_4_)_2_ salt was dissolved in the desired volume of 25 mM HEPES buffer (pH 7.5) containing 2 mM sodium dithionite. The protein samples were made anaerobic by multiple rounds of gentle degassing and flushing with argon on ice, then adjusted to contain 2 mM sodium dithionite. All equilibrium spectroscopy studies used a 1 cm path length, 2 mL quartz cuvette fitted with a stopper and purged with argon. Samples were transferred into the cuvette using a gastight syringe (Hamilton) that had been flushed with 2 mM dithionite buffer. Difference spectra were recorded for samples to which anaerobic aliquots (10 μL) of Fe­(NH_4_)_2_(SO_4_)_2_, 2OG, and l-Arg had been added, blanking against the enzyme and dithionite mixture.

### Enzyme Assays

Enzyme assays were performed at room temperature (22 ± 1 °C unless noted otherwise) in 10 mm × 16 mm tubes (BD Vacutainer Serum). Aliquots of EFE (using varied amounts as noted in the text or figure legends) were incubated in 2 mL of 25 mM HEPES buffer (pH 7.5) containing the indicated concentrations of 2OG, l-Arg, Fe­(NH_4_)_2_(SO_4_)_2_, and l-ascorbic acid. The reactions were vortexed then terminated at designated time points by adding 0.1 mL of 0.3 M HCl unless mentioned otherwise. Ethylene formation was measured by withdrawing 0.25 mL of the headspace with a Hamilton gastight syringe and injecting it into a gas chromatograph (Shimadzu GC-8A) equipped with a flame ionization detector and a Porapak N-packed column (80/100 mesh, 2 m × ^1^/_8_ inch) in an oven set at 80 °C and using N_2_ as the carrier gas. The instrument was calibrated using known concentrations of ethylene (SCOTTY Analyzed Gases, 99.5%).

The concentrations of P5C or δ^1^-piperideine-6-carboxylate (P6C) were determined by one of three approaches depending on the required sensitivity. When large amounts of the product was formed, an aliquot (1 mL) of the reaction mixture was mixed with 0.2 mL of 10 mM 2-aminobenzaldehyde in 40% ethanol, incubated at 37 °C for 20 min to develop the yellow adduct, and the absorbance was measured at 440 nm using an extinction coefficient of 2.58 mM^–1^ cm^–1^.^36^ Alternatively, the reactions were terminated with 0.1 mL of 3.6 M HCl, and 1 mL samples were derivatized by adding 0.2 mL of 2% ninhydrin in water. The mixtures were then heated to 100 °C for ∼ 30 min, cooled, and centrifuged at 10,500 *g* for 15–30 min at 4 °C. After decanting the supernatant, the reddish-brown sediment was resuspended in ethanol (0.5 mL), vortexed to extract the P5C-ninhydrin chromogen (a pinkish color), and transformed to a bluish color by adding 0.5 mL of 50 mM Tris-HCl buffer (pH 8.0). Following centrifugation at 7,500 *g* for 10 min, the absorbance at 620 nm was measured and the concentration of P5C was calculated based on the established molar extinction coefficient of 1.96 mM^–1^ cm^–1^ for the P5C–ninhydrin adduct.^37^ As a less laborious but similarly sensitive option, 2.8 mL of ninhydrin reagent (0.15% in glacial acetic acid) was added to 0.4 mL of sample, mixed, incubated at room temperature for 45 min, and the absorbance versus a reagent blank was recorded at 430 nm.^38^

The production of 3HP, succinic acid and 2-OG were quantified by liquid chromatography-tandem mass spectrometry (LC-MS/MS).^39,40^ Reaction mixtures and standards (100 μL) were quenched with three volumes of acetonitrile followed by adding 50 μL of methanol and 100 μL of dried pyridine to 100 μL of the quenched mixture. The samples were rocked gently at 25% speed in a Reliable Scientific Rocker for 10–20 min followed by addition and mixing of 30 μL 1-ethyl-3-(3-dimethyaminopropyl)-carbodiimide (EDC) solution (13.6 mg mL^–1^) in methanol-dried pyridine (20:80 v/v) and 50 μL of 4-bromo-*N*-methylbenzylamine solution (4.8 mM in dried pyridine). The tubes were maintained at 72 °C for 45 min or until the organic solvents evaporated. The samples were dissolved in 150 μL of a solution containing 2 parts of 0.1% formic acid in water and one part of acetonitrile along with 350 μL of ethyl acetate. After mixing in a rocker for 20 min, the samples were centrifuged at ∼ 2,500 *g* for 10 min and the supernatants were transferred to new Eppendorf tubes. After drying under a stream of N_2_ or by SpeedVac, the samples were reconstituted into 100 μL of water containing 0.1% formic acid and methanol (1:1 v/v). The analyte was analyzed by electrospray ionization-mass spectrometry (ESI-MS) using a XEVO G2-XS instrument in positive ionization mode. MassLynx 4.2 software (Waters, Milford, MA, USA) was used for data acquisition and analysis. The autosampler injected 10 μL of sample maintained at a compartment temperature of 10 °C. Analytes were injected onto a Waters Acquity premier BEH C18 (2.1 × 100 mm) column that was equilibrated in 10 mM ammonium formate in water, pH 2.8, at 40 °C and eluted with an increasing gradient of acetonitrile at a flow rate of 0.3 mL/min. The total run time per injection was 10 min.

### Crystallization and Data Analysis

For crystallization, TEV-cleaved Anc357 was further purified by size exclusion chromatography using a Superdex HiLoad 16/600 75 prep grade column (GE Healthcare Life Sciences). The column was equilibrated with 25 mM HEPES, pH 8.0, containing 100 mM NaCl and 1 mM tris­(2-carboxyethyl)­phosphine (TCEP). The protein was concentrated and buffer exchanged into 25 mM HEPES buffer, pH 8.0, supplemented with 1 mM TCEP, and concentrated to ∼ 40 mg/mL using an Amicon ultracentrifugation unit (molecular-weight cutoff 10,000 Da). Crystallization was performed in 96 well plates by the sitting drop vapor diffusion technique and using a mosquito crystallization robot (TTP Labtech). Initial crystallization conditions were explored using Index HT (Hampton Research), Crystal Screen HT (Hampton Research), Wizard 1&2 (Rigaku Reagents), and Wizard 3&4 (Rigaku Reagents). Crystals grew in a single condition containing 25% PEG 3350, 100 mM HEPES (pH 7.5), and 200 mM sodium chloride at 4 °C. The crystals of Anc357 apoprotein were retrieved using a nylon loop and soaked in 25% poly­(ethylene glycol) monomethyl ether (550 MME) 75% reservoir solution containing 1 mM MnCl_2_ before flash freezing in liquid nitrogen.

X-ray diffraction data were collected at the Advanced Photon Source LS-CAT beamline 21-ID-D. For details see Table S3. Data sets were indexed and integrated with iMosflm^41^ and merging and scaling were done using Aimless.^42^ Molecular replacement used *Pa*IPNS (PDB: 6JYV)^27^ as the search model. The initial model was generated using Phaser^43^ and yielded a solution with a translational function Z-score (TFZ) = 13.5 and log-likelihood gain (LLG) = 407. Additional cycles of model building were done in COOT^44^ and refinement was done in Phenix,^45^ with gradual inclusion of solvent molecules. Individual B-factors and translation, rotation, screw-rotation (TLS) refinement produced a final R_work_ of 19% (R_free_ = 22%). The resolution cutoff was determined by CC_1/2_.^46^ Data sets were uploaded to the PDB with ID 9OVH.

## Results and Discussion

### Analysis of an EFE-like IPNS Family Oxygenase

The structure of *Pa*IPNS (PDB: 6JYV) was previously described,^27^ but its function is unknown. The antibiotic-producing IPNS enzymes are related in sequence and structure to the Fe­(II)/2OG-dependent oxygenases even though they do not utilize 2OG as a cosubstrate during their catalytic transformation of ACV into isopenicillin N,^47^ a precursor of penicillin and cephalosporin.^48^ In contrast, *Pa*IPNS does not bind ACV and is unable to accommodate this peptide in its active site.^27^ Because the *Pa*IPNS structure exhibits the signature jelly roll fold and coordinates an iron ion using a strictly conserved 2-His-1-carboxylate motif, it is likely to be a member of this broad Fe­(II)/2OG-dependent oxygenase superfamily.^11^

We searched for structural similarity of *Pa*IPNS to other enzymes using DALI^49^ and identified closest similarity to PK2 EFE (PDB ID: 5V2Z, Z-score 31.7; RMSD of 2.3 Å for all 287 aligned residues). The sequence alignment of these two proteins revealed 22% identity (Figures S2 and S3). Super positioning of the active site structure for *Pa*IPNS with that of the 2OG-, l-Arg-, and metal-bound PK2 EFE (PDB: 5V2Y) revealed identical residues for binding the metal and for interactions with the carboxylates of 2OG (Figure 2A), whereas significant decreases in hydrophobicity were noted in the 2OG binding pocket (e.g., Phe175, Ala279, and Ala281 of PK2 EFE were replaced by Tyr183, Ser270, and Pro272 in *Pa*IPNS, as also highlighted in the alignment of Figure S2) and large changes at the l-Arg binding site (Figure 2B & C). For PK2 EFE, l-Arg interacts directly with Arg316 and is positioned near Met313, Phe314, and Cys317. In contrast, the corresponding residues in *Pa*IPNS are Lys324, Lys321, Val322, and Val325. Furthermore, Glu84, Val85, and Thr86 of PK2 EFE are replaced in *Pa*IPNS by Gly89, Glu90, and Leu91, which also are shifted considerably in position (Figure 2B). The l-Arg binding region has a more open helical conformation (7.5 Å from Cα of Lys321 to Cα of Lys324) compared to PK2 (Figure 2C). These changes are likely to perturb the binding of both 2OG and l-Arg by *Pa*IPNS in comparison to PK2 or Pd1 EFE; however, further evidence was required to investigate this conjecture.

**Figure 2:**
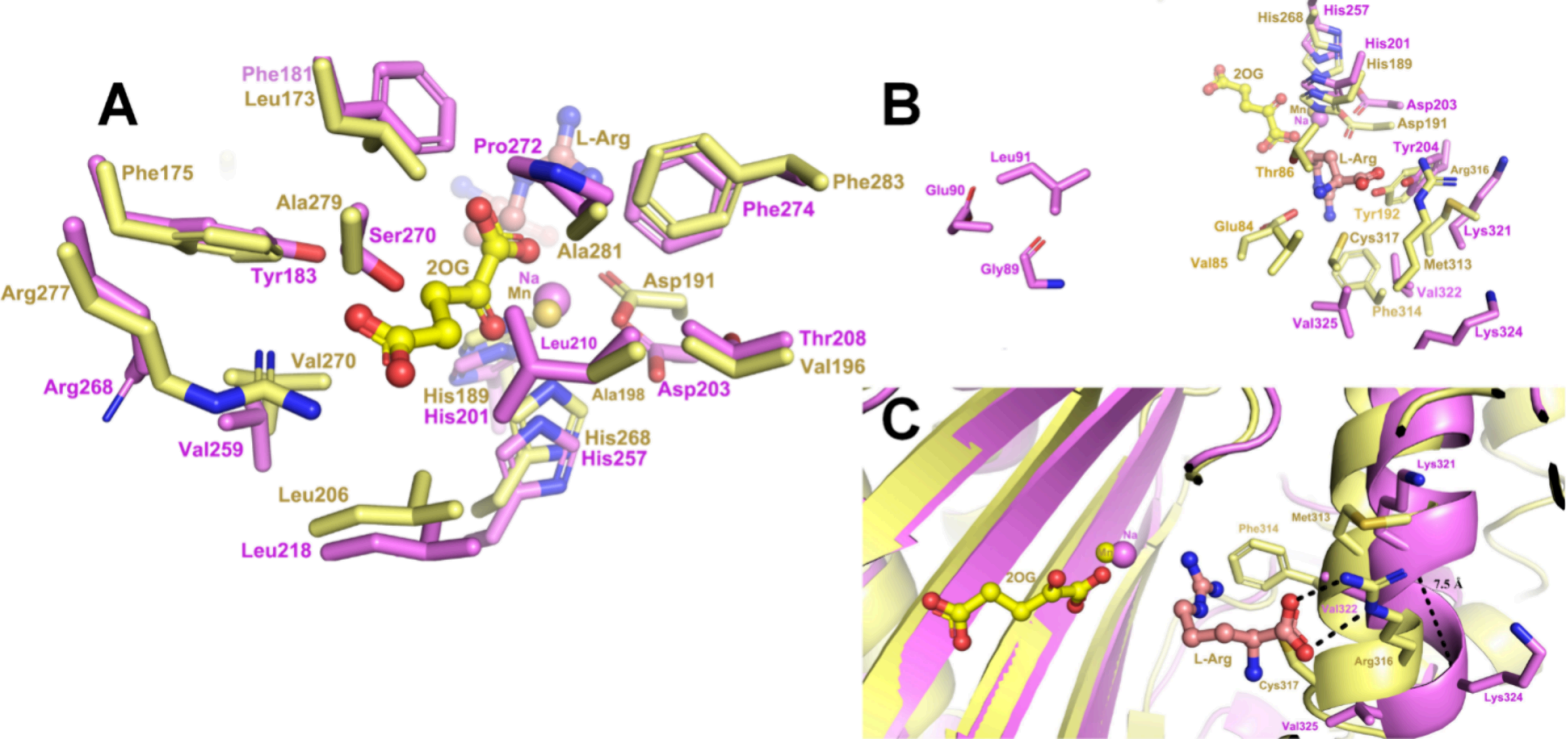
Views comparing the active sites of PK2 EFE·Mn­(II)·2OG·l-Arg (PDB ID: 5V2Y, yellow carbon atoms) and *Pa*IPNS·Na (PDB ID: 6JYV, magenta carbons). The views emphasize potential interactions with (A) 2OG and (B & C) l-Arg. Both panels show residues in stick view with N atoms in blue, O atoms in red, and with Mn or Na shown as spheres of the respective color. Panel C also depicts protein regions in cartoon mode.

We tested whether 2OG and l-Arg could bind to *Pa*IPNS by using anaerobic difference UV–visible spectroscopy (Figure 3). Starting with an anaerobic solution of *Pa*IPNS containing Fe­(II), we showed that the addition of 2OG gave rise to a difference spectrum with a λ_max_ of ∼ 530 nm and an extinction coefficient of ∼ 127 M^–1^ cm^–1^, consistent with chelation of the metal ion by the 2-keto acid to generate metal-to-ligand charge transfer (MLCT) transitions as noted for many members of the 2OG-dependent oxygenase family.^50,51^ Subsequent addition of l-Arg resulted in a diminished difference spectrum with a λ_max_ of 530 nm and an extinction coefficient of ∼ 72 M^–1^ cm^–1^, possibly suggesting that l-Arg weakly competes with 2OG binding. This behavior differs from that of PK2 EFE for which 2OG addition leads to a much smaller MLCT feature at 515 nm (∼28 M^–1^ cm^–1^), interpreted as a mixed population of monodentate and bidentate binding, that undergoes a significant increase in intensity upon binding l-Arg (to ∼ 79 M^–1^ cm^–1^).^19^ It also differs from the difference spectra of Pd1 EFE for which the 2OG-bound species exhibits maximal absorbance near 600 nm (165 M^–1^ cm^–1^) and is slightly enhanced upon l-Arg addition (178 M^–1^ cm^–1^).^13^ The dihedral angle between the C2 carbonyl and C1 carboxyl groups of the 2-oxo acid alters the π* energy level and affects the wavelength of the absorption maximum for the major MLCT transition.^50^ The *Pa*IPNS spectra clearly demonstrated that *Pa*IPNS binds 2OG, with a perturbed dihedral angle compared to that in PK2 and Pd1 EFEs, whereas the situation is less certain for the binding of l-Arg.

**Figure 3:**
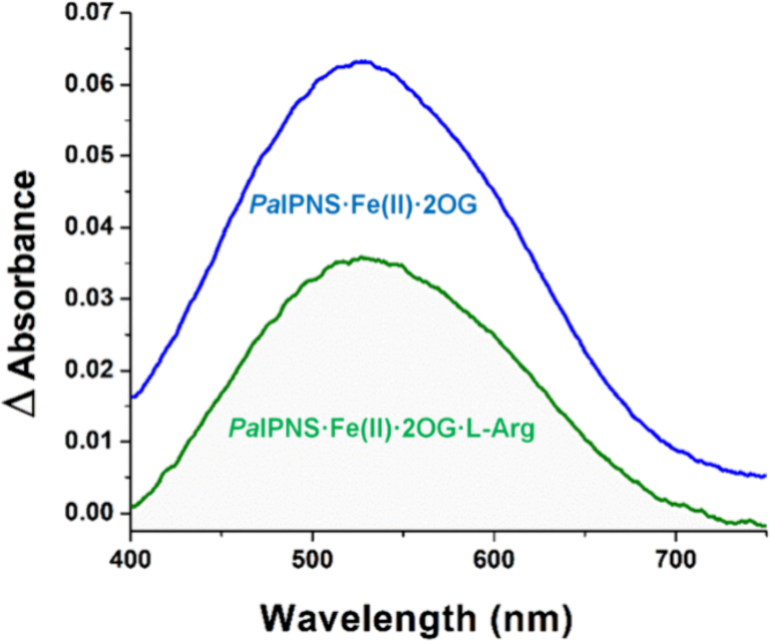
Difference absorbance spectra of *Pa*IPNS demonstrates the binding of 2OG to the active site of the protein. Spectra are shown for *Pa*IPNS·Fe­(II)·2OG (blue) and *Pa*IPNS·Fe­(II)·2OG·l-Arg (green) complexes, with the spectrum of PaIPNS·Fe­(II) taken as the blank. The anaerobic samples contained 500 μM *Pa*IPNS, 2 mM sodium dithionite, 1 mM Fe­(NH_4_)_2_(SO_4_)_2_, 1 mM 2OG, and (when present) 1 mM l-Arg in 25 mM HEPES buffer, pH 7.5. The pH of l-Arg and 2OG solutions were adjusted to 7.5 prior to degassing.

To directly test whether the enzyme exhibited any of the EFE catalytic functions, we incubated *Pa*IPNS (∼200 μM) in 0.3 mL of 25 mM HEPES buffer (pH 7.5) with 6.67 mM 2OG, 6.67 mM l-Arg, 0.4 mM Fe­(II), and 0.8 mM ascorbate for ∼ 120 min at room temperature (22 ± 1 °C). Trace levels of ethylene and 3HP (1.30 ± 0.01 nmoles and 0.5 ± 0.1 nmoles, respectively, from 2000 nmoles of 2OG) were detected by gas chromatography and derivatization was followed by ESI-MS, respectively, as shown in row 3 of Table 1 (which also shows the activities of PK2 and Pd1 EFEs, Din11, and the predicted ancestral proteins). Moreover, we demonstrated by derivatization/ESI-MS that *Pa*IPNS produced ∼700 or ∼1800 times more succinic acid (930 ± 100 nmoles) than either ethylene or 3HP, respectively, under these reaction conditions, supporting the presence of uncoupled oxidative decarboxylation of 2OG. In contrast, no P5C was detected using the chromophoric assay.

**Table 1:** Analysis of EFE-Like Catalytic Activities by *Pa*IPNS, Din11, and Ancestral Proteins^a^

	Low Protein Concentration^b^		High Protein Concentration^c^				
Protein Sample	Ethylene (nmol)	P5C (nmol)	Ethylene (nmol)	P5C (nmol)	Succinate (nmol)	3HP (nmol)	2OG (nmol)
PK2 EFE	570 ± 60	167 ± 17	1210 ± 70	350 ± 60	300 ± 10	10 ± 1	50 ± 4
Pd1 EFE	565 ± 56	97 ± 10	1380 ± 110	222 ± 23	490 ± 45	16 ± 2	9 ± 1
*Pa*IPNS	ND^d^	ND	1.30 ± 0.01	ND	930 ± 100	0.5 ± 0.1	800 ± 150
Din11	ND	ND	4.8 ± 0.1	27 ± 5	60 ± 3	2.0 ± 0.3	1970 ± 80
Anc124	ND	ND	0.3 ± 0.1	ND	1230 ± 60	0.5 ± 0.1	805 ± 140
Anc317	ND	ND	0.9 ± 0.1	ND	490 ± 70	0.6 ± 0.1	1460 ± 20
Anc357	ND	ND	0.6 ± 0.1	ND	520 ± 25	0.6 ± 0.1	1425 ± 20
Node 3	680 ± 70	236 ± 9	1110 ± 10	440 ± 30	720 ± 50	15 ± 3	3.0 ± 0.9
Node 5	810 ± 130	345 ± 43	1260 ± 100	490 ± 10	740 ± 110	11.0 ± 0.1	1.0 ± 0.1
Node 10	758 ± 15	311 ± 9	1061 ± 2	445 ± 33	780 ± 100	10 ± 1	4.0 ± 0.5
Node 13	590 ± 40	263 ± 23	1108 ± 8	480 ± 50	750 ± 30	11 ± 1	1.5 ± 0.9
Node 253	57 ± 1	9 ± 2	823 ± 1	170 ± 20	1020 ± 120	17 ± 1	11 ± 5
Node 326	250 ± 20	65 ± 5	900 ± 80	217 ± 6	1200 ± 200	18 ± 2	70 ± 20
Node 384	28 ± 3	1.7 ± 1.9	361 ± 6	20 ± 14	900 ± 1	15.0 ± 0.1	220 ± 80
Node 385	1.5 ± 0.1	1.3 ± 6	33.0 ± 0.2	17 ± 10	150 ± 40	35 ± 2	1800 ± 100

a Using diluted (columns 2-3) or concentrated (columns 4-8) proteins, the production of ethylene and P5C was assessed by gas chromatography and colorimetric assays, respectively, and for the concentrated protein samples the levels of succinate and 3HP along with the amount of remaining 2OG were analyzed by LC-MS/MS.

b Diluted enzymes (∼250–280 nM) were incubated for 80 min at room temperature (22 ± 1 °C) in 2 mL of 25 mM HEPES buffer (pH 7.5) containing 1 mM 2OG (a total of 2000 nmol), 1 mM l-Arg, 0.4 mM Fe­(NH_4_)_2_(SO_4_)_2_, and 1 mM l-ascorbate. The values were derived from two biological replicates, each having three technical replicates.

c Concentrated enzymes (∼200 μM) were incubated for 120 min at room temperature in 0.3 mL of 25 mM HEPES buffer (pH 7.5) with 6.67 mM 2OG (also a total of 2000 nmol), 6.67 mM l-Arg, 0.4 mM Fe­(NH_4_)_2_(SO_4_)_2_, and 0.8 mM l-ascorbate, then quenched with 0.9 mL acetonitrile. The values were derived from at least duplicate analyses of one biological replicate.

d ND, not detected or below the limit of detection. Standard deviations are indicated.

These results demonstrate that the extant *Pa*IPNS does not function as an EFE under the conditions tested, as evidenced by its low-level production of ethylene and 3HP and lack of detectable P5C. However, the protein’s structural similarity to PK2 and Pd1 EFEs, combined with the presence of trace EFE-like activity, suggest that *Pa*IPNS and EFE may have descended from a common ancestral enzyme with modest ethylene-forming capacity. This ancestral enzyme could represent an evolutionary precursor to modern EFEs that underwent functional specialization to efficiently catalyze ethylene generation.

### Analysis of Din11

Homoarginine is accumulated to high concentrations in seeds of *Lathyrus* species,^52,53^ providing a potential environmental nitrogen source for microorganisms. *A. thaliana* plants synthesize three *At*HA6H enzymes that transform l-homoarginine, 2OG, and O_2_ into succinate, CO_2_, and 6-hydroxy-l-homoarginine, which spontaneously decomposes to guanidine and 2-amino-6-semialdehyde that cyclizes to P6C.^54^ The *At*HA6H named Din11 also exhibits arginine 5-hydroxylase activity. That study reported no ethylene was generated by *E. coli* cultures producing any of the three enzymes.

Because the sequence of Din11 is 18% and 19% identical in sequence to those of PK2 and Pd1 EFEs (Figures S2 and S3) we assessed the ability of this protein to carry out each of the EFE-like activities using the same conditions cited above (Table 1). Contrary to the prior report, we showed that trace amounts of ethylene (4.8 ± 0.1 nmoles) were produced by Din11 incubated with 2000 nmoles of 2OG. As previously reported,^54^ this enzyme hydroxylated l-Arg to produce P5C (27 ± 5 nmoles). In addition, we found that the protein catalyzed the uncoupled oxidative decarboxylation of 2OG to produce succinate (60 ± 3 nmoles) and the conversion of 2OG to 3HP (2.0 ± 0.3 nmoles). When provided l-homoarginine as substrate, Din11 produced 0.6 ± 0.1 nmoles ethylene and 25 ± 3 nmoles P6C along with 217 ± 19 nmoles of succinate.

The superposition of the AlphaFold2 ^55^ model of Din11 and the PK2 EFE·Mn­(II)·2OG·l-Arg structure (PDB: 5V2Y) revealed similarity with some differences in the 2OG-binding site, retaining most of the hydrophobic residues while substituting Tyr217, Thr238, and Cys301 for PK2 EFE residues Phe175, Val196, and Ala281 (Figures 4 & S2). In contrast, the residues involved in l-Arg binding differ greatly between the two structures. Notably, the guanidinium group of Arg316 in PK2 EFE, which directly interacts with the carboxylate of l-Arg, is replaced by Thr348 in Din11. Furthermore, Met313, Phe314, and Cys317 of PK2 EFE are substituted with Lys345, Val346, and Thr349 in Din11. The demonstration that ethylene is produced by Din11 suggests that a precursor of this protein also was an ancestor of EFEs. This hypothesis led us to carry out a more detailed analysis of the properties of potential EFE ancestors.

**Figure 4:**
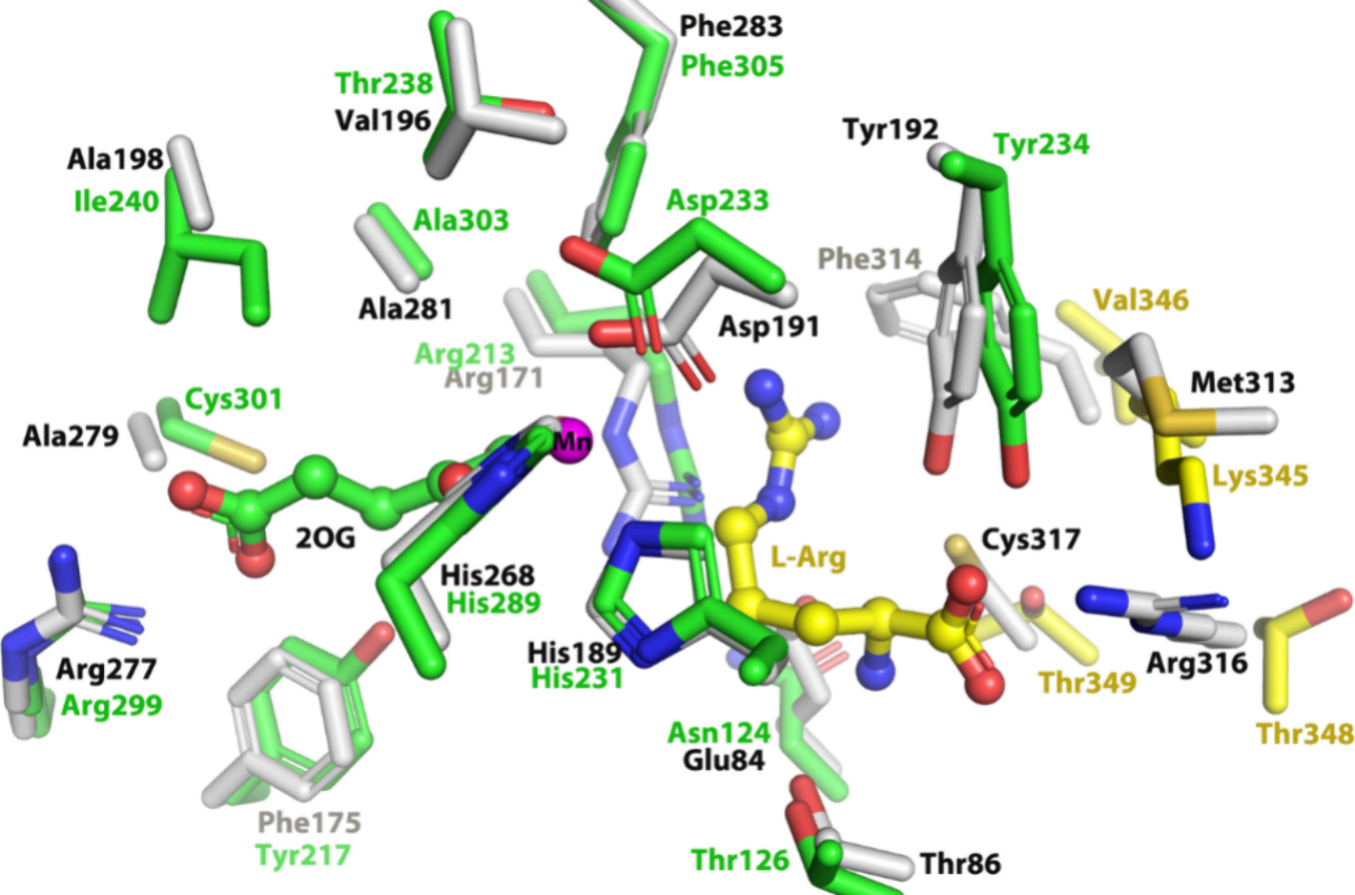
Structural overlap of PK2 EFE·Mn­(II)·2OG·l-Arg (gray) (PDB: 5V2Y) and the Din11 model. 2OG and residues of Din11 presumed to be near this substrate are indicated in green, while l-Arg and residues likely to be present at its binding site are shown in yellow. The side chains are displayed in stick representation whereas 2OG and l-Arg are represented using a ball and stick model. Nitrogen (N) atoms are in blue and oxygen (O) atoms in red, and Mn ion is shown as a magenta sphere.

### Ancestral Sequence Reconstruction (ASR) of EFE

To better understand the origins of the divergent functions of EFEs, we used ASR to predict several potential ancestral proteins for characterization. The use of ASR has recently proven useful in identifying the evolution for other biocatalytic reactions, such as those of the P450 taxadiene hydroxylase, flavin-containing monooxygenases, and 2OG-dependent nonheme iron-containing oxygenases.^56–59^ We anticipate that this tool will extend our understanding of sequence features which control the dominant reaction pathways of EFE-related enzymes.

We utilized two different approaches to select potential precursors to the extant EFEs. Using MEGA X^31^ we identified three potential ancestor sequences (denoted Anc124, Anc317, and Anc357). The Anc357 and Anc317 sequences are quite closely related to that of *Pa*IPNS, whereas the Anc124 sequence is more like those of PK2 and Pd1 EFEs (Figure 5 & Figure S2). In addition we used AP-LASR^32^ to select eight high-quality ancestors (Nodes 3, 5, 10, 13, 253, 326, 384, and 385) with ultrafast bootstrap values >95% and SH-aLRT supports of >80% (Table S1) (Figure S1). The selected ancestor node sequences exhibit varied similarities to *Pa*IPNS, Din11, and the Pd1 and PK2 EFE sequences (Figure 5 & Figure S2) with the sequence identities indicated (Figure S3). For example, the sequences of proteins corresponding to Nodes 253 and 326 are 73% and 58% identical to Pd1 EFE and the sequence of the protein corresponding to Node 10 is 80% identical to that of PK2 EFE. All the selected ancestral sequences retain the Fe­(II)-coordinating and 2OG-binding residues, but there are differences in the l-Arg binding sites as well as in the secondary coordination spheres and longer distances from the metallocenter.

**Figure 5:**
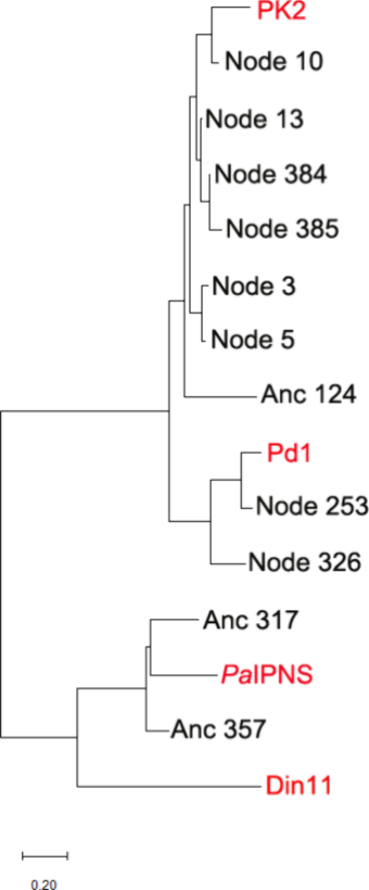
Phylogenetic analysis indicates the sequence relationships among the extant Pd1 EFE, PK2 EFE, *Pa*IPNS, and Din11 sequences, along with the ancestral sequences generated from both MEGA X and AP-LASR. The maximum likelihood phylogenetic tree was constructed using the Jones-Taylor-Thornton model and the CLUSTALW alignment plugin in MEGA11.^60^ Gaps and missing data were eliminated using pairwise deletion, and bootstrap analysis used 1000 replicates. A scale bar indicating the number of substitutions per site is shown at the bottom.

### General Characterization of Ancestral Proteins

All selected ancestor proteins were soluble and able to be purified by Ni-NTA chromatography (illustrated for ancestral proteins associated with nodes in Figure S4); however, the Anc317 protein appeared to be toxic to the *E. coli* cells resulting in poor growth, so only small quantities of it were able to be obtained. Detailed size and spectroscopic analyses were carried out for the Anc124 and Anc357 proteins. As purified, these His_6_-tagged samples exhibited dual ESI-MS features (*m*/*z* 40,614 and 40,792 for Anc124; *m*/*z* 39,352 and 39,530 for Anc357) (Figure S5) that were consistent with the expected sizes of the proteins missing their initiator methionine residues along with masses that were increased by 178 Da which we attribute to *in vivo* glycosylation of the amino terminus.^61^ TEV protease treatment removed the His_6_ tags and the glycosylation adduct to produce single species; e.g. the final Anc357 protein (*m*/*z* 37,416) was of the expected size (*m*/*z* 37,413). Size exclusion chromatography was used to demonstrate the Anc124 and Anc357 samples were monomeric (estimated as 38.7 and 52.5 kDa, respectively). Anaerobic solutions containing Fe­(II) and Anc124 or Anc357 exhibited difference spectra upon the addition of 2OG (λ_max_ of ∼ 520 nm with ε_520 nm_ of ∼ 169 M^–1^ cm^–1^ and ∼ 108 M^–1^ cm^–1^, respectively), consistent with 2OG binding (Figure S6). The subsequent addition of l-Arg resulted in diminished difference spectra (ε_520_ of ∼ 150 M^–1^ cm^–1^ and ∼ 74 M^–1^ cm^–1^, respectively), as was noted previously when using *Pa*IPNS. Solutions containing the Anc357 protein, as well as proteins associated with Nodes 5, 13, and to a lesser extent 3, were blue in color, consistent with tight binding of nickel ion from the affinity column as previously reported for PK2 EFE. Similar to the previous report for Pd1 EFE,^13^ the solutions containing proteins corresponding to Anc124, Anc317, and the other nodes were yellow in color indicating a weaker affinity for nickel ions with some retention of ferric ions. Following treatment with EDTA and buffer exchange, all samples were shown to lack metal ions when analyzed by ICP-MS. The apoprotein samples were utilized in assays containing Fe­(II) ions to investigate the EFE-like activities of the ancestral proteins.

### Activities of the Ancestral Proteins

We incubated each protein ancestor at low concentration (∼250–280 nM) in assay buffer containing 2000 nmoles of 2OG for 80 min and examined the amounts of ethylene and P5C generated (Table 1, columns 2 and 3). Using these conditions, no ethylene or P5C was detected for Anc124, Anc317, or Anc357, whereas all proteins inferred by AP-LASR were active to varying extents. Repeating the assays at high enzyme concentrations (∼200 μM) for 120 min, the production of ethylene, P5C, succinate, and 3HP were assessed as well as the remaining 2OG (Table 1, columns 4–8). In general, the levels of ethylene, P5C, and 3HP were positively correlated with the sequence identities to the PK2 and Pd1 enzymes, whereas the production of succinate did not exhibit such a correlation (Figure S7). Trace levels of ethylene were detected for Anc124, Anc317 and Anc357 proteins for these conditions, but P5C remained undetectable. These results closely resemble what was noted for *Pa*IPNS to which the Anc317 and Anc357 sequences are closely related (Figures 5, Figures S2 & S3). The near lack of activity for the Anc124 protein is somewhat surprising because its sequence is more like both the PK2 and Pd1 enzymes. Although these three ancestor proteins are essentially ineffective for ethylene generation and l-Arg hydroxylation, they did exhibit substantial succinate formation by oxidative decarboxylation of 2OG and, more interestingly, they formed detectable amounts of 3HP, again like *Pa*IPNS.

The PK2 EFE sequence corresponds most closely to the ancestral protein associated with Node 10 (Figures 5, Figures S1–S3). As expected, the activities of the Node 10 protein closely resemble those of the well-studied bacterial EFE. Indeed, the ethylene/P5C partition ratio of the Node 10 protein (2.4 ± 0.1 or 2.4 ± 0.2, depending on the protein concentration) agrees reasonably well with a previous report (3.8 ± 0.7)^13^ and with the newly acquired data (3.4 ± 0.7 and 3.5 ± 0.8 for the two conditions) using PK2 EFE (Table 1, Figure 6). The next closest ancestor of PK2 EFE is the protein associated with Node 13 (Figure 5, Figures S1 & S2). The partition ratio of this protein (2.2 ± 0.3 and 2.3 ± 0.3) also resembles the PK2 EFE ratio (Figure 6). Ancestral proteins associated with Nodes 3 and 5 also share extensive sequence similarities with PK2 EFE (Figure 5, Figures S1 & S2), and their activities are comparable (Table 1). The partition ratio of these proteins (2.9 ± 0.4 and 2.5 ± 0.2 for the two studies of the Node 3 protein and 2.4 ± 0.7 and 2.6 ± 0.3 for the Node 5 protein) also resembles that of PK2 EFE (Figure 6). The proteins associated with Nodes 384 and 385 surprisingly also fall within this sequence group (Figure 5), but their production of ethylene and P5C is severely diminished. These are among the most distant ancestor sequences examined (Figure 5 & Figure S1). The low activities of these proteins are accompanied by large error values, but the protein associated with Node 384 is of special interest because of its large partition ratio (18 ± 13 for the more concentrated enzyme sample, Figure 6), consistent with a strong preference for forming ethylene compared to l-Arg hydroxylation. In contrast, the enzyme associated with Node 385 exhibited the more typical ratio (1.9 ± 1.1 for the concentrated protein).

**Figure 6:**
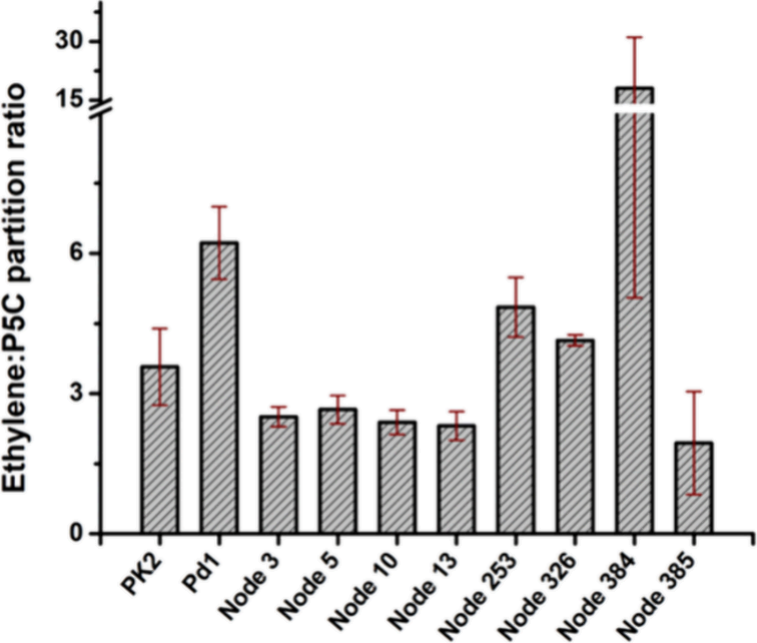
Analysis of the ethylene-to-P5C partition ratios for PK2 EFE, Pd1 EFE, and selected reconstructed ancestors, based on the studies using concentrated protein samples. The error bars represent standard deviations from at least two technical replicates.

Pd1 EFE is most closely related to the ancestor corresponding to Node 253 and less so to that of the Node 326 associated protein (Figure 5, Figures S1–S3). The ethylene and P5C forming activities of the Node 253 sample (Table 1) provides a partition ratio (6.3 ± 1.5 and 4.8 ± 0.6 for the two protein concentration conditions, Figure 6) that is highly reminiscent of Pd1 EFE (5.8 ± 1.2 and 6.2 ± 1.1).^13^ The analogous enzyme activities for the more ancestral Node 326 protein (Table 1) provided an intermediate value of the partition ratio (3.8 ± 0.6 and 4.1 ± 0.5, for the two protein concentrations) (Figure 6).

The above results reveal that ancestral proteins which retain activity exhibit only modest changes in the ethylene-to-P5C partition ratio. This finding is difficult to reconcile with the notion that EFE enzymes evolved from an ancestor which possessed singular activity either for transforming 2OG into ethylene or for hydroxylating l-Arg (Figure 1 **A** **&** [Fig fig1] **B**). Thus, a tentative conclusion is that the ancestor of EFE already was a bifunctional enzyme capable of performing both reactions. On the other hand, the proteins associated with Nodes 253, 326, 384, and 385 generated succinate levels that were 6-, 5.5-, 45-, and 8.8-fold greater than the amounts of P5C that were produced. This enhanced oxidative decarboxylation of 2OG may indicate a propensity of the ancestral enzyme to transform a yet-to-be-identified substrate or to limit cellular 2OG levels. Another possibility, based on the production of 3HP by all nodal proteins, is that the ancestor functioned in 3HP biosynthesis.

### Structural and Sequence-Based Comparison of Ancestral and Modern Sequences

We successfully crystallized the Anc357 protein in complex with Mn­(II) and solved the structure of the complex at a resolution of 2.1 Å in the P2_1_ space group (Table S3) using *Pa*IPNS (PDB: 6JYV)^27^ as a template for molecular replacement. The Anc357 protein is a monomer in solution, however the asymmetric unit contains two molecules (chains A and B) with an overall Cα RMSD of 0.2 Å. We were unable to model several disordered regions of the Mn­(II)-bound Anc357 structure (chain A residues 1–7, 190–198, 220–224, 261–263, 292–304 and chain B residues 1–8, 189–199, 223–224, 261–264, 289–310). The Anc357 protein structure (Figure 7A) includes a double-stranded β-helix (DSBH, also known as the jellyroll or cupin fold) core,^62^ an architecture that is widely found in members of the Fe­(II)/2OG-dependent oxygenases. The metal-binding site of the Anc357 protein resembles what is found in most other Fe­(II)/2OG-dependent oxygenases, with the metal coordinated by His200 and Asp202, that derive from the loop linking β5 and β6, along with His257 that is situated at the N-terminus of β10 (Figure 7B).

**Figure 7:**
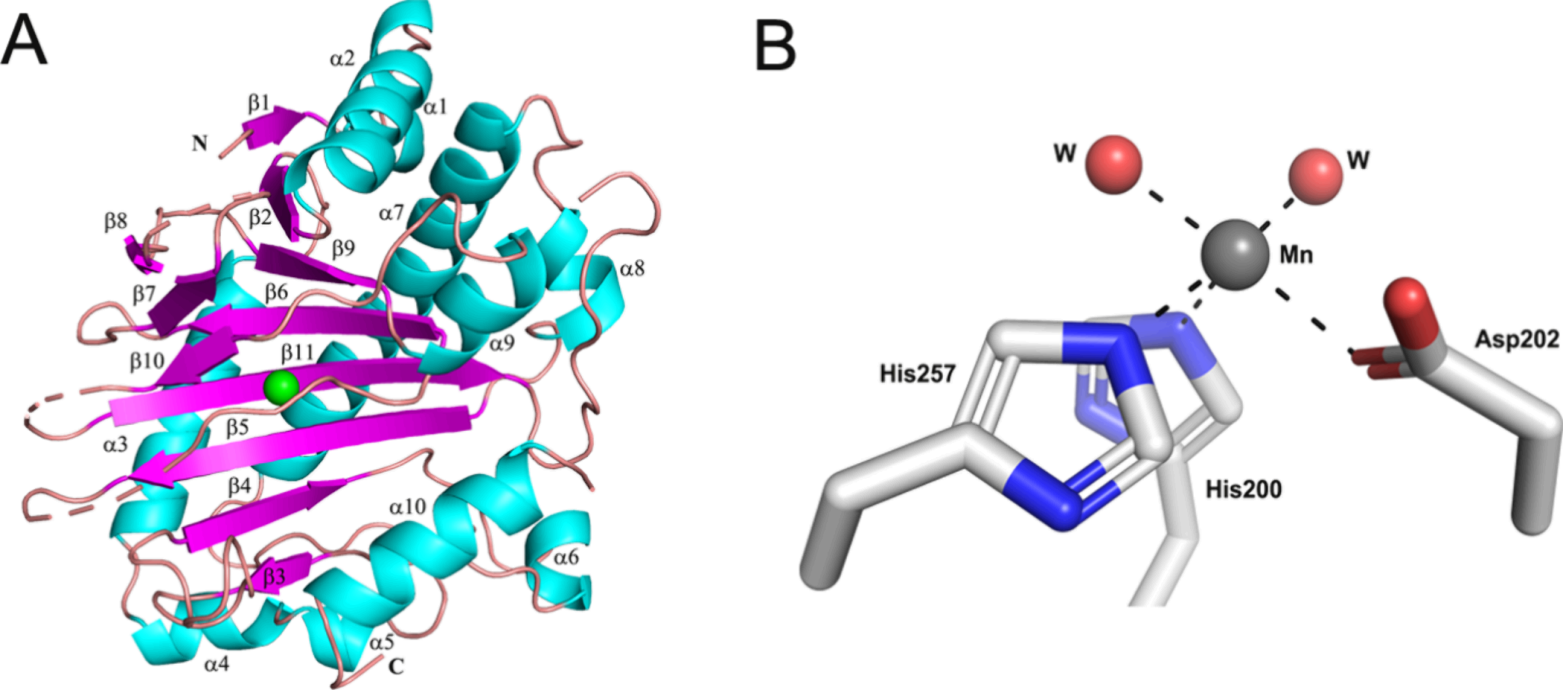
Overall fold and metal binding site of the Anc357 protein. (A) Structure of Anc357·Mn­(II) in cartoon view using chain A. Mn­(II) is shown as a green sphere. β-strands are shown in pink, α-helices in cyan, and other regions in salmon color. (B) Metal binding site of the Anc357·Mn­(II) complex.

An overlap between PK2 EFE·Mn­(II)·2OG·l-Arg and Anc357·Mn­(II) shows a Cα RMSD of 1.4 Å and reveals that Fe and 2OG binding residues for this ancestor match the situation for PK2 EFE (Figure 8A). Similarly, the Anc357 residues maintain much of the hydrophobic environment of the 2OG binding pocket (Figure 8B), although Pro93, Phe175, and Ala279 in PK2 EFE are replaced by Lys100, Tyr185, and Ser269 of the Anc357 protein. In contrast, however, there are large differences in the l-Arg binding site (Figure 8B & C). The orientation of Arg181 differs from that of Arg171 in PK2 EFE·Mn­(II)·2OG·l-Arg, presumably because this sample did not include 2OG which helps to position the guanidino group. Of greater significance, the l-Arg binding residue Arg316 of PK2 EFE aligns with Lys323 in the Anc357 protein. The side chains of Lys323 and Arg267 in Anc357 were not visible due to their greater flexibility and may interfere with substrate binding. The Anc357 loop between β7 and β8, spanning residues 220 to 226 (shown in yellow, Figure 8A), is significantly shorter than the PK2 EFE β11 loop, residues 209 to 237 (shown in red, Figure 8A). In PK2 EFE, this loop contains Glu215 (shown as red stick view), a residue that is crucial for ethylene and P5C formation, as its substitution by alanine leads to a decrease in these products.^20^ Recently, the significance of Glu215 was highlighted as a long-range residue that shields the active site from solvent exposure.^63^ The greatly shortened loop and changes at the l-Arg binding site of the Anc357 protein compared to PK2 EFE explain why the enzyme is essentially inactive.

**Figure 8:**
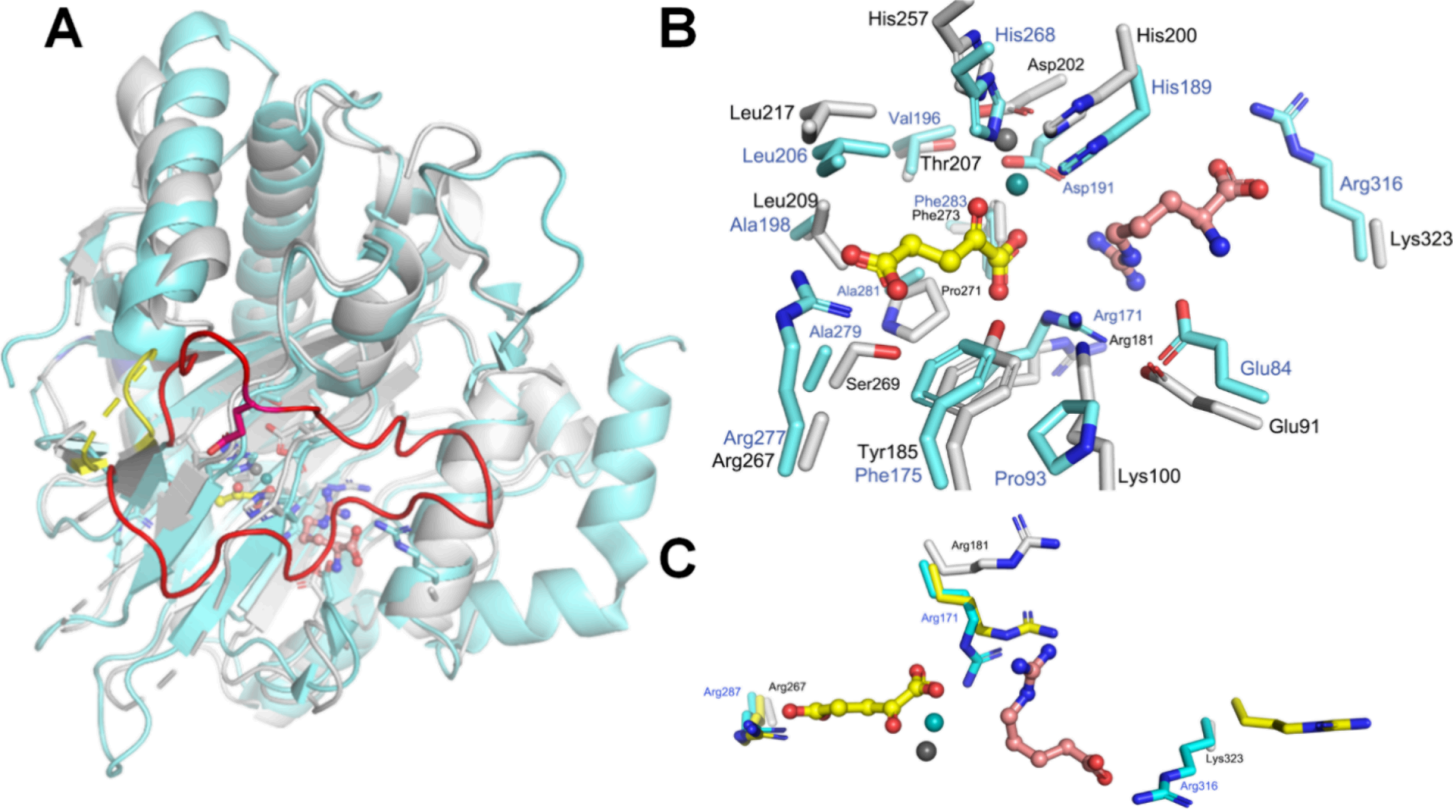
Superposition of Anc357·Mn­(II) and PK2 EFE·Mn­(II)·2OG·l-Arg (PDB: 5V2Y). (A) The Anc357 protein is gray and PK2 EFE with bound 2OG and l-Arg is cyan, both shown in cartoon mode with selected components depicted as sticks. A short loop in the Anc357 protein (yellow) substitutes for a much longer loop in PK2 EFE (red). (B and C) Active site comparisons with 2OG in yellow and l-Arg in salmon, both depicted in ball-and-stick mode, the PK2 EFE Mn­(II) as a teal sphere, and the Anc357 Mn­(II) shown as a gray sphere. Panel C also shows the altered positions of Arg171 in the PK2 EFE apoprotein (PDB: 5V2U, yellow). The flexibility of PK2 EFE Arg171 does not extend to the modeled position of Arg181 in the Anc357 protein.

To gain additional structural insights into the functional properties of Anc124, Anc317, and the ancestral node proteins, for which crystal structures are not available, we generated and examined their AlphaFold2 ^55^ models as the next best recourse. Notably, comparing an AlphaFold2 model of the Anc357 protein (which is among the ancestral sequences with least sequence similarity to PK2 EFE, Figure S3) to the newly solved structure reveals an RMSD of ∼ 0.44 Å, so the models are likely to predict the structures of other proteins possessing the EFE fold with high accuracy. Nevertheless, caution should be used when interpreting AlphaFold-predicted structures.^64^

An overlay of the model for the Anc124 protein with the PK2 EFE·Mn­(II)·2OG·l-Arg structure reveals near identity in their folds, but with several changes affecting the 2OG binding site (Figure S8). Whereas PK2 EFE has residues Phe175, Ala198, and Ala279, the Anc124 protein possesses Tyr176, Thr199, and Ser280 at the corresponding positions leading to a more hydrophilic 2OG environment of reduced size. We previously noted that, other than its carboxylate-binding interactions, 2OG binds to PK2 EFE in a pocket lined by hydrophobic residues,^20^ so the indicated changes in Anc124 likely account for the greatly reduced activity of this reconstructed ancestor. Succinate production by this protein (Table 1) confirms that this protein does bind 2OG.

A similar overlay of the Anc317 model with the EFE structure (Figure S9) highlights the 23-residue shortened β11 loop compared to PK2 EFE, analogous to what was noted in the Anc357 protein. Multiple additional changes are present at both the 2OG and l-Arg binding sites. Replacing Phe175, Val196, and Ala279 lining the 2OG pocket of PK2 EFE are the more hydrophilic residues Tyr189, Thr213, and Ser274 in the ancestral protein. Moreover, Leu173, Ala198, and Ala281 in PK2 EFE are replaced by the bulkier residues Phe187, Leu215, and Pro276 of Anc317. Also, Met313, Phe314, Arg316, and Cys317 near the l-Arg binding site of PK2 EFE are replaced by Lys325, Val326, Lys328, and Val329 residues with quite different properties in the ancestral protein. Numerous additional changes are found in more distant sites of the proteins (Figure S9).

In contrast to the multiple changes noted for the above reconstructed ancestors, single changes were present at the active sites for the proteins corresponding to Nodes 3, 5, 10, and 13 sequences (Figure S10). For proteins associated with Nodes 3, 5, and 13, Met281 substitutes for Cys280 in PK2 EFE. In contrast, the Node 10 protein possesses leucine at this position. The PK2 EFE cysteinyl residue was proposed to interact with dioxygen in the tunnel that accesses the metallocenter.^65^ Analysis of C280M and C280L variants of PK2 EFE did not lead to changes in ethylene or P5C formation and consequently did not exhibit changes in the ethylene:P5C ratio (unpublished observations). The shifts in position of Arg317 in the ancestor proteins, compared to Arg316 of PK2 EFE, is attributed to the known change in position of this residue when comparing the PK2 enzyme with and without l-Arg.^20^ Other more distant residues differ among the proteins, as exemplified by the protein corresponding to Node 13 with its Phe248 residue substituted for PK2 EFE Trp247. Consistent with the near identity of the active sites in these ancestral proteins to PK2 EFE, they exhibited very similar activities (Table 1).

The protein corresponding to Node 384 had a large partition ratio (Figure 6), albeit a somewhat low activity (Table 1), so it was of special interest. A superposition of the active site for the Node 384 protein and that of PK2 EFE revealed close identity with only Met281 in the ancestor replacing Cys280 of the extant enzyme (Figure S11A). To further investigate the basis of the increased partition ratio, changes involving slightly more distant residues were examined. The cluster of Phe159, Thr163, and Leu194 residues in PK2 EFE are replaced by Leu160, Ala164, and Phe195 in the nodal protein (Figure S11B), potentially affecting the mobility or stability of this region of the enzyme which is not close to the substrate binding sites. Perhaps more importantly, the change from Phe310 in PK2 EFE to Val311 in the Node 384 protein perturbs a hydrophobic patch that includes additional phenylalanyl and tryptophanyl residues located proximal to the l-Arg binding site (Figure S11C,D). The smaller Val311 residue would provide extra room for mobility in this region as indicated by the flip in orientation of Trp168 in the modeled structure. A decrease in l-Arg binding would reduce the production of P5C and possibly account for the greater partition ratio if the amino acid substitution still allowed for ethylene generation. Thus, we speculate that the change from Phe310 in PK2 EFE to Val311 in this ancestor is potentially a contributing factor to the enhanced partition ratio.

Of interest, the Node 253 and 326 proteins possessed small changes at the l-Arg binding site compared to the PK2 EFE (Ile83 or Ile84 replacing Val85, and Asn321 or Asn323 replacing Cys317) (Figure S12). In addition, the Node 253 and 326 proteins possessed the large hydrophobic residue Phe280 or Phe282, respectively, at the position of Cys280 in PK2 EFE near the 2OG binding site. Notably, these ancestral proteins possess partition ratios like that of Pd1 EFE, which also exhibits these three substitutions.

The Node 385 protein exhibited the least activity of this group of ancestors, and the homology model provides a reasonable explanation (Figure S13). Residues Val85 and Thr86 at the PK2 EFE binding site for l-Arg are replaced by Lys85 and Lys86 in this reconstructed ancestor, the latter of which would clash with the binding of this substrate. As in several other ancestral proteins, the Cys280 residue of PK2 EFE is replaced by Met281. The reason why this enzyme generates the largest amounts of 3HP remain unclear. Nevertheless, the distinctions noted here confirm the critical role of experimentally verified key residues in PK2 that enable the ethylene-forming enzyme to produce ethylene, P5C, and succinate.

## Conclusions

Our studies of EFE-related enzymes, both distant modern homologues and ancestral proteins, have provided fresh insights into the evolution and properties of this enzyme. We found that the IPNS family oxygenase from *P. aeruginosa* is closely related in structure to PK2 EFE and generates trace levels of ethylene and 3HP, undetectable amounts of P5C, and substantial uncoupled production of succinate. Din11, a plant homoarginine 6-hydroxylase that also hydroxylates C5 of l-Arg, is 29% identical in sequence to the *Pa*IPNS protein, similarly produces small amounts of ethylene and 3HP, but generates greater levels of P5C and exhibits only small amounts of uncoupled succinate production. The *Pa*IPNS and *At*HA6H proteins possess metal-binding sites that are identical to what is found in PK2 EFE, both bind 2OG, the latter also binds l-Arg. The near absence of ethylene formation by *Pa*IPNS and Din11 can be rationalized in part by the differences in residues corresponding to the PK2 EFE l-Arg binding site.

Ancestral EFE proteins, inferred by maximum likelihood phylogeny, demonstrate similar features to the *Pa*IPNS and Din11 proteins. Specifically, the sequences of the predicted ancestral proteins Anc317 and Anc357 are 63% and 66% identical to *Pa*IPNS as well as 32% and 34% identical to Din11, whereas the Anc124 protein is only 23% and 19% identical in sequence to these proteins but closely related to PK2 (56%) and Pd1 (42%) EFEs. All three ancestral proteins generate ethylene and 3HP in trace amounts, had P5C levels below the detection limit, and formed varying uncoupled production levels of succinate, much like the catalytic properties of *Pa*IPNS and Din11. Structural modeling of the Anc124 and Anc317 proteins suggest the near absence of activity compared to PK2 EFE is due to enhanced hydrophilicity at the 2OG binding site of both proteins with multiple additional changes in the Anc317 protein affecting the steric bulk and charges at the l-Arg binding site. The crystallographically resolved structure of Anc357 reveals only small differences in the 2OG binding pocket but large differences from PK2 EFE in the l-Arg binding site, accounting for its depressed activity levels.

The reconstructed proteins corresponding to Nodes 3, 5, 10, and 13 are most closely related in sequence to PK2 EFE and exhibit very similar activities and partition ratios. Proteins corresponding to the Node 253 and 326 sequences are most related to Pd1 EFE but exhibit somewhat reduced activities for ethylene formation and P5C production while retaining analogous partition ratios, and form 3HP at similar amounts. Of great interest, the Node 384 and Node 385 proteins, which correspond to the most distant of the reconstructed ancestors, show rather poor ability for generating ethylene or P5C, form substantial levels of 3HP, and exhibit the most extreme partition ratios—very large for the Node 384 protein and low for the enzyme corresponding to Node 385.

Overall, the results presented here refute the notion that the original ancestor of extent EFEs possessed a single activity for either ethylene formation or l-Arg hydroxylation. Rather, we find that all the active ancestors retained both activities as if the two activities are inherent to catalysis. Of added interest, however, some of the ancestral proteins exhibited high levels of uncoupled 2OG decarboxylation consistent with their functioning either in another oxygenase reaction or as a mechanism to limit cellular 2OG levels. In addition, it is interesting to note that the most distant of the reconstructed ancestors generated the greatest levels of 3HP, raising the remote possibility of that process being relevant to the function of the primordial enzyme. This work also highlights the importance of residues at the l-Arg pocket for ethylene formation, emphasizing the need for additional engineering efforts involving this region.

## Supplementary Material



## Notes

Ancestral sequence reconstruction tree, sequence alignment and identity matrix for proteins described here, SDS-PAGE of purified ancestral proteins, ESI-MS and anaerobic difference UV–visible spectra of selected samples, correlation plots of enzyme products versus sequence identities, analyses of AlphaFold2 models of protein ancestors, confidence values for EFE ancestral nodes, primer sequences, and crystallographic data and refinement statistics (PDF)

## Notes

UniProt accession codes for EFEs from Pd1 and PK2 are A0A7T7BQH3 and P32021, that for *Pa*IPNS is Q9HWJ0, and that for Din11 is Q8H113.

## 6

Department of Biochemistry Molecular Biology and Biophysics, University of Minnesota, Minneapolis, Minnesota 55108, United States

## 7

Department of Chemical and Biomolecular Engineering, University of Delaware, Newark, Delaware 19713, United States
