# Supplementary material for: Ancestral Sequence Reconstruction of the Ethylene-Forming Enzyme
Source: Biochemistry. 2025 Jul 25;64(15):3432–45. doi: 10.1021/acs.biochem.5c00334 (PMC12329707; doi:10.1021/acs.biochem.5c00334)
Supplement: Supplementary file 1 [file bi5c00334_si_001.pdf]

Supporting Information for

Ancestral Sequence Reconstruction of the Ethylene-Forming Enzyme

Shramana Chatterjee,[1] Joel A. Rankin,[1,6] Mark A. Farrugia,[1] Bryce J. Delaney,[1] Nathaniel S. Pascual,[2,3] James VanAntwerp,[2,3,7] Daniel R. Woldring,[2,3,*] Jian Hu,[4,5,*] and Robert P. Hausinger[1,4,*]

[1]Department of Microbiology, Genetics, and Immunology, Michigan State University, East Lansing, Michigan 48824, United States

[2]Department of Chemical Engineering and Materials Science, Michigan State University, East Lansing, Michigan 48824, United States

[3]Institute for Quantitative Health Science and Engineering, Michigan State University, East Lansing, Michigan 48824, United States

[4]Department of Biochemistry and Molecular Biology, Michigan State University, East Lansing, Michigan 48824, United States

[5]Department of Chemistry, Michigan State University, East Lansing, Michigan 48824, United States

[6]Present Address: Department of Biochemistry Molecular Biology and Biophysics, University of Minnesota, Minneapolis, Minnesota 55108, United States

[7]Present Address: Department of Chemical and Biomolecular Engineering, University of Delaware, Newark, Delaware 19713, United States

*To whom correspondence should be addressed: woldring@msu.edu, hujian1@msu.edu, hausinge@msu.edu

A

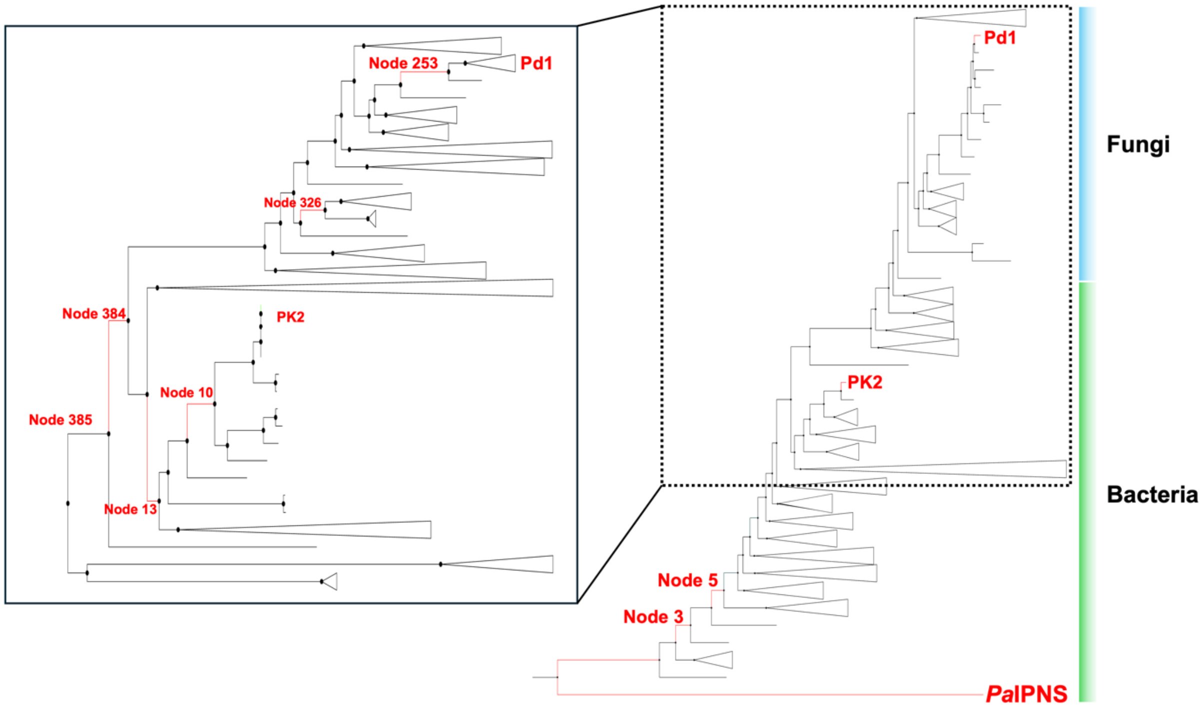
Node 253
Pd1
Node 326
Node 384
PK2
Node 10
Node 385
Node 13
Pd1
Fungi
PK2
Bacteria
Node 5
Node 3
PaIPNS

B

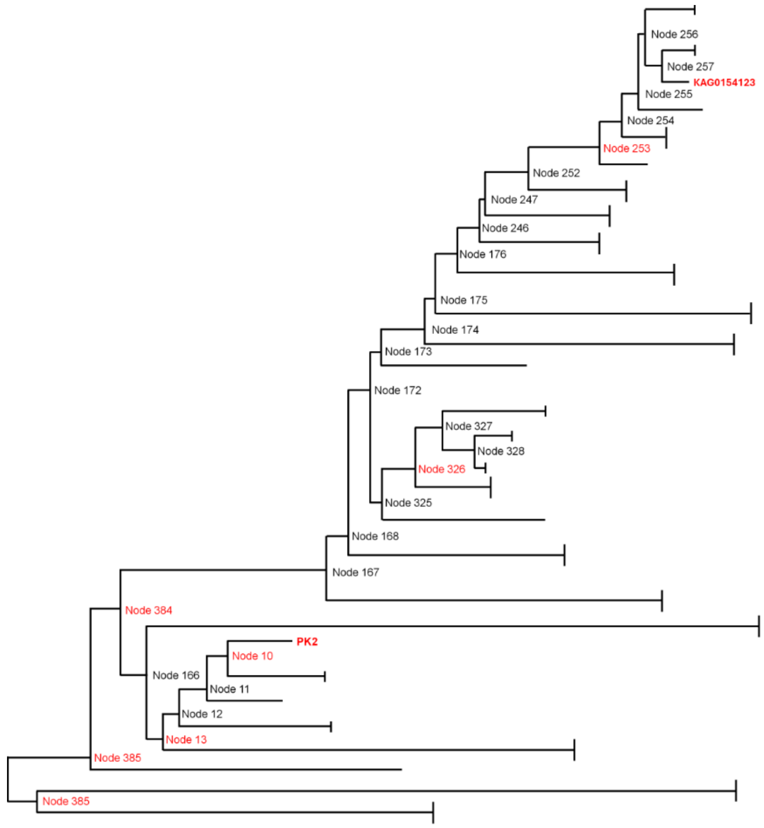
Node 256
Node 257
KAG0154123
Node 255
Node 254
Node 253
Node 252
Node 247
Node 246
Node 176
Node 175
Node 174
Node 173
Node 172
Node 327
Node 328
Node 326
Node 325
Node 168
Node 167
Node 384
PK2
Node 10
Node 166
Node 11
Node 12
Node 13
Node 385
Node 385

S2

C

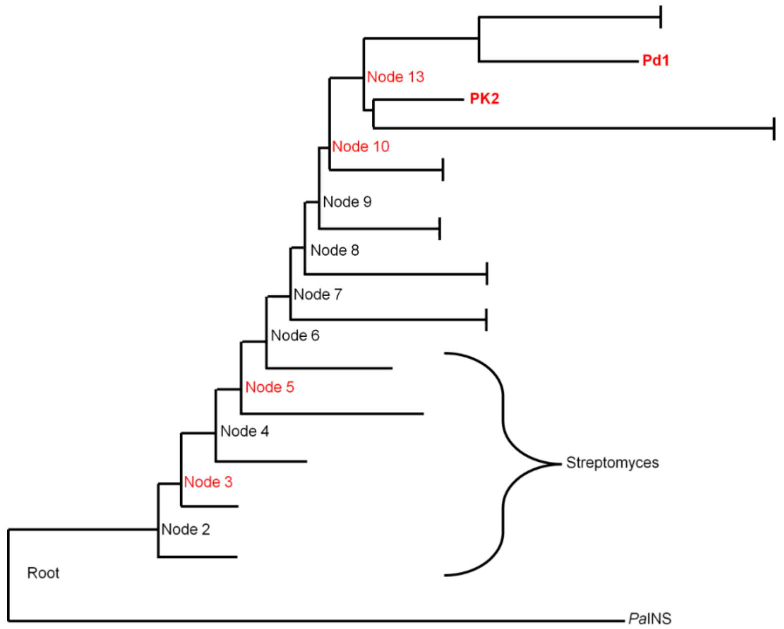

Figure S1. AP-LASR derived ancestral sequence reconstruction trees. (A) Abbreviated combined tree showing the positions of the extant PK2 and Pd1 EFEs and highlighting Nodes 3, 5, 10, 13, 253, 326, 384, and 385 that were further characterized here (the nodes of interest refer to the branches below and to the right of the labels except for Node 13 which refers to the branch above and to the right). (B) Abbreviated initial tree with labels shown to the right of the nodes. (C) Abbreviated tree created using *Pa*IPNS as an outgroup with labels shown to the right of the nodes. Average confidence values for the nodes of interest are shown in **Table S1**. The two completely annotated tree files are provided in the following two pages.

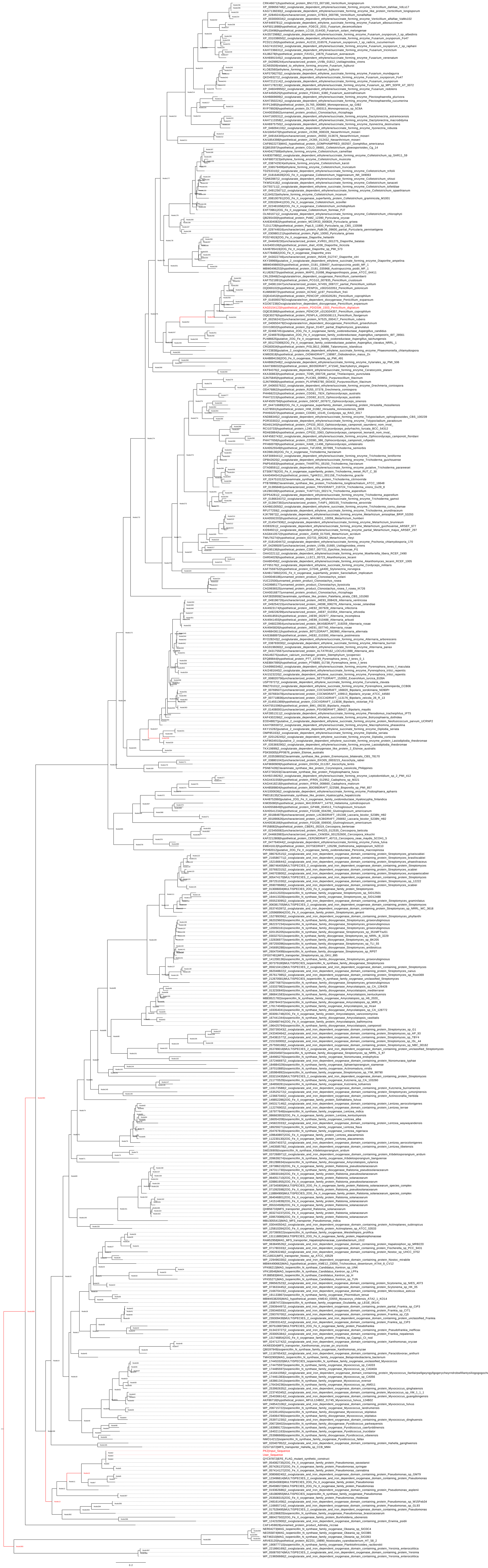
0.2

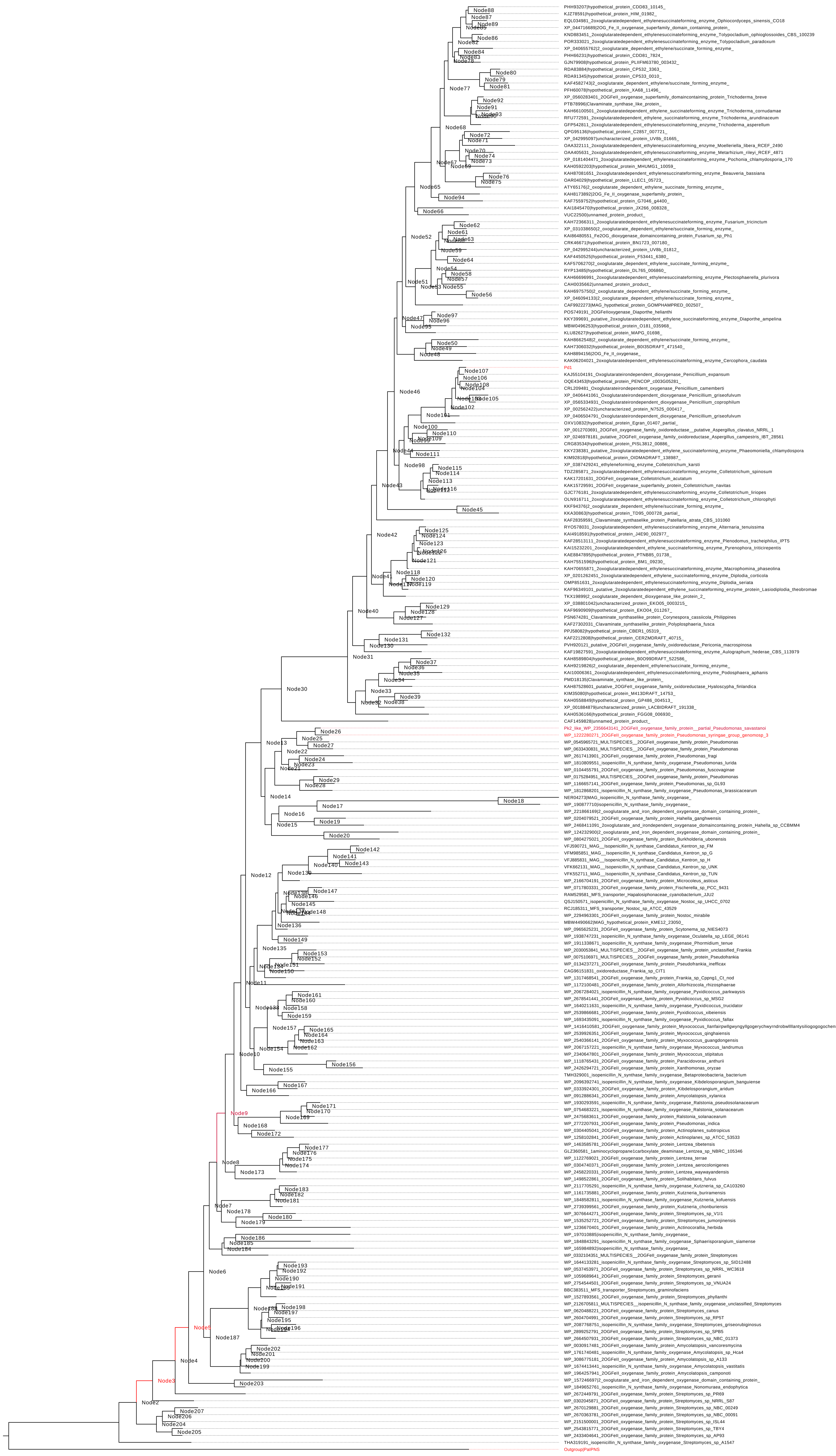
PHH93207|hypothetical_protein_CDD83_10145_
KJZ78591|hypothetical_protein_HIM_01982_
EQL034981_2oxoglutaratedependent_ethylenesuccinateforming_enzyme_Ophiocordyceps_sinensis_CO18
XP_044716689|2OG_Fe_II_oxygenase_superfamily_domain_containing_protein_
KND883451_2oxoglutaratedependent_ethylenesuccinateforming_enzyme_Tolypocladium_ophioglossoides_CBS_100239
POR333021_2oxoglutaratedependent_ethylenesuccinateforming_enzyme_Tolypocladium_paradoxum
XP_040655762|2_oxoglutarate_dependent_ethylene/succinate_forming_enzyme_
PHH66231|hypothetical_protein_CDD81_7824_
GJN79908|hypothetical_protein_PLIIFM63780_003432_
RDA83884|hypothetical_protein_CP532_3363_
RDA91345|hypothetical_protein_CP533_0010_
KAF4582743|2_oxoglutarate_dependent_ethylene/succinate_forming_enzyme_
PFH60078|hypothetical_protein_XA68_11496_
XP_0560283401_2OGFeII_oxygenase_superfamily_domaincontaining_protein_Trichoderma_breve
PTB78996|Clavaminate_synthase_like_protein_
KAH66100501_2oxoglutaratedependent_ethylene_succinateforming_enzyme_Trichoderma_cornudamae
RFU772591_2oxoglutaratedependent_ethylenesuccinateforming_enzyme_Trichoderma_arundinaceum
GFP542811_2oxoglutaratedependent_ethylenesuccinateforming_enzyme_Trichoderma_asperellum
QPG95136|hypothetical_protein_C2857_007721_
XP_042995097|uncharacterized_protein_UV8b_01665_
OAA322111_2oxoglutaratedependent_ethylenesuccinateforming_enzyme_Moelleriella_libera_RCEF_2490
OAA405631_2oxoglutaratedependent_ethylenesuccinateforming_enzyme_Metarhizium_rileyi_RCEF_4871
XP_0181404471_2oxoglutaratedependent_ethylenesuccinateforming_enzyme_Pochonia_chlamydosporia_170
KAH0592203|hypothetical_protein_MHUMG1_10059_
KAH87081651_2oxoglutaratedependent_ethylenesuccinateforming_enzyme_Beauveria_bassiana
OAR04029|hypothetical_protein_LLEC1_05723_
ATY65176|2_oxoglutarate_dependent_ethylene_succinate_forming_enzyme_
KAH8173892|2OG_Fe_II_oxygenase_superfamily_protein_
KAF7559752|hypothetical_protein_G7046_g4400_
KAI1845470|hypothetical_protein_JX266_008328_
VUC22500|unnamed_protein_product_
KAH72366311_2oxoglutaratedependent_ethylenesuccinateforming_enzyme_Fusarium_tricinctum
XP_031038650|2_oxoglutarate_dependent_ethylene/succinate_forming_enzyme_
KAI86480551_Fe2OG_dioxygenase_domaincontaining_protein_Fusarium_sp_Ph1
CRK46671|hypothetical_protein_BN1723_007180_
XP_042995244|uncharacterized_protein_UV8b_01812_
KAF4450525|hypothetical_protein_F53441_6380_
KAF5706270|2_oxoglutarate_dependent_ethylene_succinate_forming_enzyme_
RYP13485|hypothetical_protein_DL765_006860_
KAH66696991_2oxoglutaratedependent_ethylenesuccinateforming_enzyme_Plectosphaerella_plurivora
CAH0035662|unnamed_protein_product_
KAH6975750|2_oxoglutarate_dependent_ethylene/succinate_forming_enzyme_
XP_046094133|2_oxoglutarate_dependent_ethylene/succinate_forming_enzyme_
CAF9922273|MAG_hypothetical_protein_GOMPHAMPRED_002507_
POS749191_2OGFeIIoxygenase_Diaporthe_helianthi
KKY399691_putative_2oxoglutaratedependent_ethylene_succinateforming_enzyme_Diaporthe_ampelina
MBW0496253|hypothetical_protein_O181_035968_
KLU82627|hypothetical_protein_MAPG_01698_
KAH8662548|2_oxoglutarate_dependent_ethylene/succinate_forming_enzyme_
KAH7306032|hypothetical_protein_B0I35DRAFT_471540_
KAH8894156|2OG_Fe_II_oxygenase_
KAK06204021_2oxoglutaratedependent_ethylenesuccinateforming_enzyme_Cercophora_caudata
Pd1
KAJ55104191_Oxoglutarateirondependent_dioxygenase_Penicillium_expansum
OQE43453|hypothetical_protein_PENCOP_c003G05281_
CRL209481_Oxoglutarateirondependent_oxygenase_Penicillium_camemberti
XP_0406441061_Oxoglutarateirondependent_dioxygenase_Penicillium_griseofulvum
XP_0565334931_Oxoglutarateirondependent_dioxygenase_Penicillium_coprophilum
XP_002562422|uncharacterized_protein_N7525_000417_
XP_0406504791_Oxoglutarateirondependent_dioxygenase_Penicillium_griseofulvum
OXV10832|hypothetical_protein_Egran_01407_partial_
XP_0012703691_2OGFeII_oxygenase_family_oxidoreductase__putative_Aspergillus_clavatus_NRRL_1
XP_0246978181_putative_2OGFeII_oxygenase_family_oxidoreductase_Aspergillus_campestris_IBT_28561
CRG83534|hypothetical_protein_PISL3812_00886_
KKY238381_putative_2oxoglutaratedependent_ethylene_succinateforming_enzyme_Phaeomoniella_chlamydospora
KIM92818|hypothetical_protein_OIDMADRAFT_138987_
XP_0387429241_ethyleneforming_enzyme_Colletotrichum_karsti
TDZ285871_2oxoglutaratedependent_ethylenesuccinateforming_enzyme_Colletotrichum_spinosum
KAK17201631_2OGFeII_oxygenase_Colletotrichum_acutatum
KAK15729591_2OGFeII_oxygenase_superfamily_protein_Colletotrichum_navitas
GJC776181_2oxoglutaratedependent_ethylenesuccinateforming_enzyme_Colletotrichum_liriopes
OLN916711_2oxoglutaratedependent_ethylenesuccinateforming_enzyme_Colletotrichum_chlorophyti
KKF94376|2_oxoglutarate_dependent_ethylene/succinate_forming_enzyme_
KKA30863|hypothetical_protein_TD95_000728_partial_
KAF28359591_Clavaminate_synthaselike_protein_Patellaria_atrata_CBS_101060
RYO578031_2oxoglutaratedependent_ethylenesuccinateforming_enzyme_Alternaria_tenuissima
KAI4918591|hypothetical_protein_J4E90_002977_
KAF28513111_2oxoglutaratedependent_ethylenesuccinateforming_enzyme_Plenodomus_tracheiphilus_IPT5
KAI15232201_2oxoglutaratedependent_ethylenesuccinateforming_enzyme_Pyrenophora_triticirepentis
KAE8847895|hypothetical_protein_PTNB85_01738_
KAH7551596|hypothetical_protein_BM1_09230_
KAH70655871_2oxoglutaratedependent_ethylenesuccinateforming_enzyme_Macrophomina_phaseolina
XP_0201262451_2oxoglutaratedependent_ethylene_succinateforming_enzyme_Diplodia_corticola
OMP851631_2oxoglutaratedependent_ethylenesuccinateforming_enzyme_Diplodia_seriata
KAF96349101_putative_2oxoglutaratedependent_ethylene_succinateforming_enzyme_protein_Lasiodiplodia_theobromae
TKX19899|2_oxoglutarate_dependent_dioxygenase_like_protein_2_
XP_038801042|uncharacterized_protein_EKO05_0003215_
KAF9690909|hypothetical_protein_EKO04_011267_
PSN674281_Clavaminate_synthaselike_protein_Corynespora_cassiicola_Philippines
KAF27302031_Clavaminate_synthaselike_protein_Polyplosphaeria_fusca
PPJ58082|hypothetical_protein_CBER1_05319_
KAF2212808|hypothetical_protein_CERZMDRAFT_40715_
PVH920121_putative_2OGFeII_oxygenase_family_oxidoreductase_Periconia_macrospinosa
KAF19827591_2oxoglutaratedependent_ethylenesuccinateforming_enzyme_Aulographum_hederae_CBS_113979
KAH8589804|hypothetical_protein_B0O99DRAFT_522586_
KAH9219826|2_oxoglutarate_dependent_ethylene/succinate_forming_enzyme_
KAI10006361_2oxoglutaratedependent_ethylenesuccinateforming_enzyme_Podosphaera_aphanis
PMD18135|Clavaminate_synthase_like_protein_
KAH87528601_putative_2OGFeII_oxygenase_family_oxidoreductase_Hyaloscypha_finlandica
KIM35080|hypothetical_protein_M413DRAFT_14753_
KAH0558849|hypothetical_protein_GP486_004513_
XP_001884879|uncharacterized_protein_LACBIDRAFT_191338_
KAH0536166|hypothetical_protein_FGG08_006930_
CAF1459828|unnamed_protein_product_
Pk2_like_WP_2356643141_2OGFeII_oxygenase_family_protein__partial_Pseudomonas_savastanoi
WP_1222280271_2OGFeII_oxygenase_family_protein_Pseudomonas_syringae_group_genomosp_3
WP_0545965721_MULTISPECIES__2OGFeII_oxygenase_family_protein_Pseudomonas
WP_0633430831_MULTISPECIES__2OGFeII_oxygenase_family_protein_Pseudomonas
WP_2617413901_2OGFeII_oxygenase_family_protein_Pseudomonas_fragi
WP_1810809551_isopenicillin_N_synthase_family_oxygenase_Pseudomonas_lurida
WP_0104455791_2OGFeII_oxygenase_family_protein_Pseudomonas_fuscovaginae
WP_0175284951_MULTISPECIES__2OGFeII_oxygenase_family_protein_Pseudomonas
WP_1166657141_2OGFeII_oxygenase_family_protein_Pseudomonas_sp_GL93
WP_1812868201_isopenicillin_N_synthase_family_oxygenase_Pseudomonas_brassicacearum
NER04273|MAG_isopenicillin_N_synthase_family_oxygenase_
WP_190877710|isopenicillin_N_synthase_family_oxygenase_
WP_221866169|2_oxoglutarate_and_iron_dependent_oxygenase_domain_containing_protein_
WP_0204079521_2OGFeII_oxygenase_family_protein_Hahella_ganghwensis
WP_2468411091_2oxoglutarate_and_irondependent_oxygenase_domaincontaining_protein_Hahella_sp_CCBMM4
WP_124232900|2_oxoglutarate_and_iron_dependent_oxygenase_domain_containing_protein_
WP_0804275021_2OGFeII_oxygenase_family_protein_Burkholderia_ubonensis
VFJ590721_MAG__Isopenicillin_N_synthase_Candidatus_Kentron_sp_FM
VFM985851_MAG__Isopenicillin_N_synthase_Candidatus_Kentron_sp_G
VFJ885831_MAG__Isopenicillin_N_synthase_Candidatus_Kentron_sp_H
VFK662131_MAG__Isopenicillin_N_synthase_Candidatus_Kentron_sp_UNK
VFK552711_MAG__Isopenicillin_N_synthase_Candidatus_Kentron_sp_TUN
WP_2166704191_2OGFeII_oxygenase_family_protein_Microcoleus_asticus
WP_0717803331_2OGFeII_oxygenase_family_protein_Fischerella_sp_PCC_9431
RAM529581_MFS_transporter_Hapalosiphonaceae_cyanobacterium_JJU2
QSJ150571_isopenicillin_N_synthase_family_oxygenase_Nostoc_sp_UHCC_0702
RCJ185311_MFS_transporter_Nostoc_sp_ATCC_43529
WP_2294963301_2OGFeII_oxygenase_family_protein_Nostoc_mirabile
MBW4490662|MAG_hypothetical_protein_KME12_23050_
WP_0965625231_2OGFeII_oxygenase_family_protein_Scytonema_sp_NIES4073
WP_1938747231_isopenicillin_N_synthase_family_oxygenase_Oculatella_sp_LEGE_06141
WP_1911338671_isopenicillin_N_synthase_family_oxygenase_Phormidium_tenue
WP_2030053841_MULTISPECIES__2OGFeII_oxygenase_family_protein_unclassified_Frankia
WP_0075106971_MULTISPECIES__2OGFeII_oxygenase_family_protein_Frankia
WP_0134237271_2OGFeII_oxygenase_family_protein_Pseudofrankia_inefficax
CAG96151831_oxidoreductase_Frankia_sp_CiT1
WP_1317468541_2OGFeII_oxygenase_family_protein_Frankia_sp_Cppng1_Ct_nod
WP_1172100481_2OGFeII_oxygenase_family_protein_Allorhizocola_rhizosphaerae
WP_2067284021_isopenicillin_N_synthase_family_oxygenase_Pyxidicoccus_parkwaysis
WP_2678541441_2OGFeII_oxygenase_family_protein_Pyxidicoccus_sp_MSG2
WP_1640211631_isopenicillin_N_synthase_family_oxygenase_Pyxidicoccus_trucidator
WP_2539866681_2OGFeII_oxygenase_family_protein_Pyxidicoccus_xibeiensis
WP_1693435091_isopenicillin_N_synthase_family_oxygenase_Pyxidicoccus_fallax
WP_1416410581_2OGFeII_oxygenase_family_protein_Myxococcus_llanfairpwllgwyngyllgogerychwyrndrobwllllantysiliogogogochensis
WP_2539926351_2OGFeII_oxygenase_family_protein_Myxococcus_qinghaiensis
WP_2540366141_2OGFeII_oxygenase_family_protein_Myxococcus_guangdongensis
WP_2067157221_isopenicillin_N_synthase_family_oxygenase_Myxococcus_landrumus
WP_2340647801_2OGFeII_oxygenase_family_protein_Myxococcus_stipitatus
WP_1118765431_2OGFeII_oxygenase_family_protein_Paracidovorax_anthurii
WP_2426294721_2OGFeII_oxygenase_family_protein_Xanthomonas_oryzae
TMH329001_isopenicillin_N_synthase_family_oxygenase_Betaproteobacteria_bacterium
WP_2096392741_isopenicillin_N_synthase_family_oxygenase_Kibdelosporangium_banguiense
WP_0333924301_2OGFeII_oxygenase_family_protein_Kibdelosporangium_aridum
WP_0912886341_2OGFeII_oxygenase_family_protein_Amycolatopsis_xylanica
WP_1930293591_isopenicillin_N_synthase_family_oxygenase_Ralstonia_pseudosolanacearum
WP_0754683221_isopenicillin_N_synthase_family_oxygenase_Ralstonia_solanacearum
WP_2475683611_2OGFeII_oxygenase_family_protein_Ralstonia_solanacearum
WP_2772207931_2OGFeII_oxygenase_family_protein_Pseudomonas_indica
WP_0304405041_2OGFeII_oxygenase_family_protein_Actinoplanes_subtropicus
WP_1258102841_2OGFeII_oxygenase_family_protein_Actinoplanes_sp_ATCC_53533
WP_1463585781_2OGFeII_oxygenase_family_protein_Lentzea_tibetensis
GLZ360581_1aminocyclopropane1carboxylate_deaminase_Lentzea_sp_NBRC_105346
WP_1122769021_2OGFeII_oxygenase_family_protein_Lentzea_terrae
WP_0304740371_2OGFeII_oxygenase_family_protein_Lentzea_aerocolonigenes
WP_2458220331_2OGFeII_oxygenase_family_protein_Lentzea_waywayandensis
WP_1498522861_2OGFeII_oxygenase_family_protein_Solihabitans_fulvus
WP_2117705291_isopenicillin_N_synthase_family_oxygenase_Kutzneria_sp_CA103260
WP_1161735881_2OGFeII_oxygenase_family_protein_Kutzneria_buriramensis
WP_1848582811_isopenicillin_N_synthase_family_oxygenase_Kutzneria_kofuensis
WP_2739399561_2OGFeII_oxygenase_family_protein_Kutzneria_chonburiensis
WP_3076644271_2OGFeII_oxygenase_family_protein_Streptomyces_sp_VII1
WP_1535252721_2OGFeII_oxygenase_family_protein_Streptomyces_jumonjinensis
WP_1236670401_2OGFeII_oxygenase_family_protein_Actinocorallia_herbida
WP_197010885|isopenicillin_N_synthase_family_oxygenase_
WP_1848843291_isopenicillin_N_synthase_family_oxygenase_Sphaerisporangium_siamense
WP_165984892|isopenicillin_N_synthase_family_oxygenase_
WP_0332104351_MULTISPECIES__2OGFeII_oxygenase_family_protein_Streptomyces
WP_1644133281_isopenicillin_N_synthase_family_oxygenase_Streptomyces_sp_SID12488
WP_0537453971_2OGFeII_oxygenase_family_protein_Streptomyces_sp_NRRL_WC3618
WP_1059689641_2OGFeII_oxygenase_family_protein_Streptomyces_gerarii
WP_2754544501_2OGFeII_oxygenase_family_protein_Streptomyces_sp_VNUA24
BBC383511_MFS_transporter_Streptomyces_graminofaciens
WP_1527893561_2OGFeII_oxygenase_family_protein_Streptomyces_phyllanthi
WP_2126705811_MULTISPECIES__isopenicillin_N_synthase_family_oxygenase_unclassified_Streptomyces
WP_0620488221_2OGFeII_oxygenase_family_protein_Streptomyces_canus
WP_2604704991_2OGFeII_oxygenase_family_protein_Streptomyces_sp_RP5T
WP_2087768751_isopenicillin_N_synthase_family_oxygenase_Streptomyces_griseorubiginosus
WP_2899252791_2OGFeII_oxygenase_family_protein_Streptomyces_sp_SPB5
WP_2664507931_2OGFeII_oxygenase_family_protein_Streptomyces_sp_NBC_01373
WP_0030917481_2OGFeII_oxygenase_family_protein_Amycolatopsis_vancoresmycina
WP_1761740481_isopenicillin_N_synthase_family_oxygenase_Amycolatopsis_sp_Hca4
WP_3086775181_2OGFeII_oxygenase_family_protein_Amycolatopsis_sp_A133
WP_1674413441_isopenicillin_N_synthase_family_oxygenase_Amycolatopsis_vastitatis
WP_1964257941_2OGFeII_oxygenase_family_protein_Amycolatopsis_camponoti
WP_157246697|2_oxoglutarate_and_iron_dependent_oxygenase_domain_containing_protein_
WP_1849652761_isopenicillin_N_synthase_family_oxygenase_Nonomuraea_endophytica
WP_2672449791_2OGFeII_oxygenase_family_protein_Streptomyces_sp_PR69
WP_0302045871_2OGFeII_oxygenase_family_protein_Streptomyces_sp_NRRL_S87
WP_2670129881_2OGFeII_oxygenase_family_protein_Streptomyces_sp_NBC_00249
WP_2670363781_2OGFeII_oxygenase_family_protein_Streptomyces_sp_NBC_00091
WP_2151500001_2OGFeII_oxygenase_family_protein_Streptomyces_sp_ISL44
WP_2543815771_2OGFeII_oxygenase_family_protein_Streptomyces_sp_TBY4
WP_2433404641_2OGFeII_oxygenase_family_protein_Streptomyces_sp_AP93
THA319191_isopenicillin_N_synthase_family_oxygenase_Streptomyces_sp_A1547
Outgroup|PalPNS

```
Node_326      ------------------------------------------------------------ 0
Pd1           -MFSHRSVS-IALRPPGLCHITFRQAYPILLTRQQTKSLTTTTAPSNLGSTMPPNYVA-R 57
Node_253      ------------------------------------------------------------ 0
Anc124        ------------------------------------------------------------ 0
PK2           ------------------------------------------------------------ 0
Node_3        ------------------------------------------------------------ 0
Node_5        ------------------------------------------------------------ 0
Node_10       ------------------------------------------------------------ 0
Node_385      ------------------------------------------------------------ 0
Node_13       ------------------------------------------------------------ 0
Node_384      ------------------------------------------------------------ 0
Din11         MVIYHRKVVFTYVRA--------KRFYHFLNIEMV----------TDFKSLLPVIDISPL 42
PsIPNS        ----------------------------------------------MSTPSLPIIDIAAL 14
Anc317        ------------------------------MTQVV----------NEEFSQIPIIDISAL 20
Anc357        ---------------------------------------------MTSMSELPVIDISPL 15


Node_326      --NLQTFELPEKITGSPGDRALGRSMIDAWRRDGILQISMDPTNRKLADAAFACSKKFFS 58
Pd1           VGQLKTFTLPETATGSPSDVELGKAMINAWREDGILQVSMSPRQQALFENASAASKRFFA 117
Node_253      ---LKTFTLPEKVTGSPGDVQMGKALINAWREDGILQIAMNPKQQDLFDKAFAASKRFFA 57
Anc124        MADFQSFHLPESISGTQSDVDLGREMIRAWRTDGAFRIAMSEIQRKKSEDAFAASRRFFR 60
PK2           MTNLQTFELPTEVTGCAADISLGRALIQAWQKDGIFQIKTDSEQDRKTQEAMAASKQFCK 60
Node_3        MTDLQTFHLPESVTGSEADIELGREMIRAWQTDGIFQVATDPAQDQKTQEAFEASRRFFR 60
Node_5        MTDLQTFHLPESVTGSEADIELGREMIQAWRTDGIFQVATDPAQDQKTQEAFEASRRFFR 60
Node_10       MTNLQTFELPETVTGSPADISLGRALIQAWRKDGIFQVATDPEQDRKTQAAMAASKRFFK 60
Node_385      MTNLQTFHLPESVTGSPADIQLGREIIQAWRKDGIIQVARDPEQDRKTQRAFEANKRFFR 60
Node_13       MTDLQTFHLPESVTGSPADIQLGRAMIQAWRKDGIFQVATDPEQDRKTQRAFEASKRFFR 60
Node_384      MTNLQTFHLPESVTGSPADIQLGRAMIQAWRKDGIFQVATDPEQDRKTQRAFEASKRFFR 60
Din11         LAKCDDFDMAE----DAGVVEVVGKLDRACRDVGFFYVIG-GISDDLINKVKEMTHQFFE 98
PsIPNS        AGSD-----------PAARRSVAVRIDRACREQGFFYVVGHGVEAQLVERLERLARQFFA 63
Anc317        VS-G-----------TGDRHTVASQIRQACRECGFFYIVGHGVDEELQQRLEQLSRQFFA 68
Anc357        VD-A-----------PG-RAEVAAQIGQACREHGFFYVTGHGVDPELQDRLEALSREFFA 62
                                   :   :  * :  * : :     .    :      :.*

Node_326      MPYKEKAKCVD---DQSFAGYIASGEEITDNIADYSEIFTVTKDLPLSDPRVQQKWPCHG 115
Pd1           MPPNQKAACVD---TQSYAGYIASGEEITDGIADYSEIFTVTKDLPLDEPRVEAKWPCHG 174
Node_253      LPPKQKAACVD---TQSYAGYIASGEEITDGIADYSEIFTVTKDLPLDEPRVKAKWPCHG 114
Anc124        GSIEFKSQYTN---DLTYSGYTASGEEVTAGERDYPEVFTICRDIPVDDARVRAHWPCHG 117
PK2           EPLTFKSSCVS---DLTYSGYVASGEEVTAGKPDFPEIFTVCKDLSVGDQRVKAGWPCHG 117
Node_3        LPLEEKARCVS---DLTYSGYIASGEEVTAGEADYSEIFTVCKDVPLDDPRVQEGWPCHG 117
Node_5        MPLEEKSRCVS---DLTYSGYIASGEEVTAGEADYSEIFTVCKDVPLDDPRVQAGWPCHG 117
Node_10       MPMEFKSSCVS---DLTYSGYIASGEEVTAGEADYSEIFTVCKDLPESDPRVKEGWPCHG 117
Node_385      RPLEEKRRYVS---DLTFSGYIASGEEKKAGEADYSEIFTVFPDLPLDDPRVKEGWPCHG 117
Node_13       MPMEEKSRCVS---DLTYSGYIASGEEVTAGEADYSEIFTVCKDLPLDDPRVKEGWPCHG 117
Node_384      RPMEEKSRYVS---DLTFSGYIASGEEVTAGEADYSEIFTVCKDLPLDDPRVKEGWPCHG 117
Din11         LPYEEKLKIKITP-TAGYRGYQRIGVNFTSGKQDMHEAIDCYREFKQG-KHGDIGKVLEG 156
PsIPNS        LDETSKLRWRMELGGRAWRGYFPLGGELTSNRPDWKEGLYLGSELDAEHPEVRAGTPLHG 123
Anc317        QDLETKLKIRMALGGRAWRGYFPVGGELTSGKPDLKEGIYFGAELEEDHPLVKAGTPLHG 128
Anc357        LPEEEKMKIRMARGGRAWRGYFPVGGELTSGKPDWKEGLYFGTELPPDHPRVRAGTPLHG 122
                   *           : **   * : . .  *  * :    :.             .*

Node_326      PCPWPN--AQMKTVMQAYMDYLGESGEKMLQLIAWGLGLPDGNALTKYTQDGWHHMRILR 173
Pd1           PCPWPD--VDMRTPIQQYMDSLGKSGETLLQMIEYGLSLHP-DTLTSLTKDGWHHLRILR 231
Node_253      PCPWPD--IDMKTPIQQYMDSLGTSGERLLQLIEYGLNLEP-KTLTSLTQDGWHHLRILR 171
Anc124        PVPWPD--AEYRESLQTYLNELGSVGDRLLQLVALGLELNDMDALLALAKDGWHHLRALR 175
PK2           PVPWPN--NTYQKSMKTFMEELGLAGERLLKLTALGFELP-INTFTDLTRDGWHHMRVLR 174
Node_3        PVPWPD--EEYQQSMKAFMDELGSIGEKLLRLIALGLGLDDIDALTRLTRDGWHHMRVLR 175
Node_5        PVPWPD--EEYQQSMKAFMDELGSIGEKLLKLTALGLGLDDIDALTKLTRDGWHHMRVLR 175
Node_10       PVPWPD--ENYQKSMKAFMDELGHIGEKLLKLVALGLELPDINTLTDLTRDGWHHMRVLR 175
Node_385      PVPWPS--EDYKEAMKAYMDELGSIGERLLQLVALGLGLPDINALTDLARDGWHHMRVLR 175
Node_13       PVPWPD--EEYRQAMKAFMDELGSIGEKLLKLVALGLGLPDINALTDLTRDGWHHMRVLR 175
Node_384      PVPWPS--EEYRQAMKAYMDELGSIGEKLLQLVALGLGLPDINALTDLARDGWHHMRVLR 175
Din11         PNQWPGNPQEYKDLMEKYIKLCTDLSRNILRGISLALGGSPYEFEGKMLRDPFWVMRIIG 216
PsIPNS        ANLFPE-VPGLRETLLEYLDATTRVGHRLMEGIALGLGLEADYFAARYTGDPLILFRLFN 182
Anc317        RNLFPSNIPQFRETVLEYMEAMTQLGHALMAGIALSLGLEESYFADRYTADPLILFRIFN 188
Anc357        RNLFPEEVPEFRETVLEYMDAMTQLGHRLMRGIALSLGLPADYFRERYTADPTVLFRIFH 182
                 :*      :  :  ::.     .  ::     .:             *    :*  :

Node_326      FPETNNTNGKGKEGRGIGSHTDYGLLVIAAQDD-VGGLFIRPPYEGEKY-ANWK--KSAA 229
Pd1           FPQNNKTNGRGKKGRGIGSHTDYGLLVIAAQDE-VGGLFIRPPADDEKL-ENWK--NSAA 287
Node_253      FPQTNNTNGKGKEGRGIGSHTDYGLLVIAAQDD-VGGLFIRPPYDDEKL-ENWK--KSAA 227
```

```
Anc124        YPVASQ-----ESNRGLGAHTDYGLIVITDEDD-VGGLYIRPPVEGEKRNRNWLADESTA 229
PK2           FPPQTS-----TLSRGIGAHTDYGLLVIAAQDD-VGGLYIRPPVEGEKRNRNWLPGESSA 228
Node_3        FPARSS-----QTARGIGAHTDYGLLVIAAQDD-VGGLYIRPPVEGEKRNRNWLPSESSA 229
Node_5        FPARSS-----KTSRGIGAHTDYGLLVIAAQDD-VGGLYIRPPVEGEKRNRNWLPGESSA 229
Node_10       FPTASS-----QSSRGIGAHTDYGLLVIAAQDD-VGGLYIRPPVEGEKRNRNWLPGESSA 229
Node_385      FPAASS-----QTSRGIGAHTDYGFLVIASQDD-VGGLYIRPPVEGEKRNRNWLPDESSA 229
Node_13       FPAASS-----QTSRGIGAHTDYGLLVIAAQDD-VGGLYIRPPVEGEKRNRNWLPGESSA 229
Node_384      FPAASS-----QTSRGIGAHTDYGFLVIAAQDD-VGGLYIRPPVEGEKRNRNWLPGESSA 229
Din11         YPGVN----Q-ENVIGCGAHTDYGLLTLINQDDDKTALQVKNV----------------- 254
PsIPNS        YPSQPVPEGL-DVQWGVGEHTDYGLLTLLHQDA-IGGLQVRTP----------------- 223
Anc317        YPPQSSPD-S-EESWGVGEHTDYGLLTILKQDD-SGGLQVKSK----------------- 228
Anc357        YPPP--PD-A-DDQWGVGEHTDYGLLTILKQDD-CGGLQVKSK----------------- 220
              :*             * * *****::.:  :*    .* ::

Node_326      GMHEDDDKWVYVPPVPDVFTVFPGDMMQYITNSYLPSTPHKVGLNT--RERFAFAYFHEP 287
Pd1           GFREDDERWVYVPPVPGVFTVFPGDIMQFMTNSYLPSTPHKVGLNT--RERFAFAYFHEP 345
Node_253      GFREDDERWVYVPPVPGVFTVFPGDMMQFMTNSYLPSTPHKVGLNT--RERYAFAYFHEP 285
Anc124        GMYENEEDWIFAKPVPNVFTVLPGDILQFITGNHILATIHKVTLNT--RERFSMAYFHEP 287
PK2           GMFEHDEPWTFVTPTPGVWTVFPGDILQFMTGGQLLSTPHKVKLNT--RERFACAYFHEP 286
Node_3        GMYENEEPWTFVKPVPSVLTVFPGDILQFLTGGRLLSTPHKVRLNT--RERFAMAYFHEP 287
Node_5        GMYENEEPWTFVKPVPSVLTVFPGDILQFLTNGYLLSTPHKVRLNT--RERFAMAYFHEP 287
Node_10       GMFENEEPWTFVKPVPKVFTVFPGDILQFMTDGYLLSTPHKVKLNT--RERFALAYFHEP 287
Node_385      GMYENEEPWTFVKPVPNVFTVFPGDMMQLLTDGYLLSTPHKVKLNT--RERFAMAYFHEP 287
Node_13       GMYENEEPWTFVKPVPNVFTVFPGDILQFLTDGYLLSTPHKVKLNT--RERFAMAYFHEP 287
Node_384      GMYENEEPWTFVKPVPNVFTVFPGDMMQLLTDGYLLSTPHKVKLNT--RERFAMAYFHEP 287
Din11         -----DGDWIPAIPIPGSFICNIGDMLTILSNGVYQSTLHKV-INNSPKYRVCVAFFYET 308
PsIPNS        ------QGWLEAPPIPGSFVCNLGDMLERMTGGLYRSTPHRVARNTSGRDRLSFPLFFDP 278
Anc317        ------SGWIDAPPIPNSFVCNIGDMLDRMTGGLYRSTPHRV-RNPSGRNRLSFPFFFDP 281
Anc357        -----GGGWIDAPPIPGTFVCNIGDMLDRMTGGLYRSTPHRV-RNTSGRDRLSFPFFFDP 274
                      *  . * *       **::  ::..   :* *:*  *   : * .   *.:

Node_326      NFSAVMKPLPGYDAGQEPTE-------------GIHYGTHFTNMFMRNYPERITANRMRS 334
Pd1           SFQAVVSPVAKLYDGQPPVE-------------KIHYGTHFTNMFMRNYPDRITTERIIK 392
Node_253      SFQAEIKPIPKLYDGQPPDE-------------KIHYGTHFTNMFMRNYPDRITTERIIR 332
Anc124        DFNACVYPLSNP----SGED-------------YLFYGEHFTNMFMRCYPDRATTRRIVD 330
PK2           NFEASAYPLFEP----SANE-------------RIHYGEHFTNMFMRCYPDRITTQRINK 329
Node_3        NFEAVVRPLSGP----DSDE-------------YIHYGTHFTNMFMRCYPDRITTRRILE 330
Node_5        NFEACVRPLSGP----DSDE-------------YIHYGTHFTNMFMRCYPDRITTRRILE 330
Node_10       NFEACARPLFDP----SSDE-------------RIHYGEHFTHMFMRCYPDRITTRRIHK 330
Node_385      NFEARVRPLNDP----SGGE-------------HIHYGEHVTNMFMRCYPDRITTRRIHE 330
Node_13       NFEACVRPLFDP----SGGE-------------HIHYGEHFTNMFMRCYPDRITTRRIHE 330
Node_384      NFEAVVRPLFDP----SGGE-------------HIHYGEHVTNMFMRCYPDRITTRRIHE 330
Din11         NFEAEVEPLDIFKEKHPRKE-------TSQVAKRVVYGQHLINKVLTTFANLVENS---- 357
PsIPNS        NFHARVQPIEGLPEVPEQDDSARRWDQANVHAFHGEYGDYLLNKVAKVFPQLRRDLL--- 334
Anc317        NFNAEVKPIEIKAGVVVNDDKEERWDKASVHEFRGTYGDYLLSKVSKVFPELRQTVLGSR 341
Anc357        NFDAEVQPLPALDRAPAEDDSAERWDGASVHAFEGTYGDYLLSKVSKVFPELREEVLE-- 332
              .* *   *:          :                **  :.   .   : :

Node_326      ENRMEMLE-------------  342
Pd1           EDRLQLLDRPELRTQ------  407
Node_253      EDRMKLLD-------------  340
Anc124        ENRLSVL--------------  337
PK2           ENRLAHLEDLKKYSDTRATGS  350
Node_3        EDRLSVLARLREAALAG----  347
Node_5        EDRLSVLARLREEALRRA---  348
Node_10       ENRLAHLEEMKQRS-------  344
Node_385      ENLLSVLEELKQ---------  342
Node_13       ENRLAVLEELRQ---------  342
Node_384      ENRLSVLEELKQ---------  342
Din11         ---------------------  357
PsIPNS        ---------------------  334
Anc317        ---------------------  341
Anc357        ---------------------  332
```

Figure S2. Sequence alignment of PK2 and Pd1 EFEs, *Ps*IPNS, Din11, Anc124, Anc317, and Anc357, along with ancestral sequence Nodes 3, 5, 10, 13, 253, 326, 384, and 385. Residues in PK2 EFE known to coordinate Fe(II) are shown in white text on a black background, those proximate to the 2OG-binding site are highlighted in green, and

those associated with the L-Arg binding site are highlighted in maroon. Differences from these residues in other sequences are indicated by gray highlights.

Protein	PK2	Pd1	Anc 317	Anc 357	Anc 124	Node 10	Node 13	Node 253	Node 326	Node 384	Node 385	Node 3	Node 5	*Pa*IPNS	Din11
**PK2**	1	0.448	0.24	0.243	0.564	0.803	0.746	0.525	0.533	0.72	0.689	0.7	0.72	0.224	0.188
**Pd1**	0.448	1	0.201	0.225	0.42	0.501	0.503	0.727	0.583	0.491	0.472	0.496	0.5	0.211	0.172
**Anc 317**	0.24	0.201	1	0.736	0.237	0.241	0.254	0.246	0.224	0.251	0.248	0.239	0.241	0.627	0.32
**Anc 357**	0.243	0.225	0.736	1	0.245	0.256	0.268	0.27	0.256	0.265	0.26	0.273	0.264	0.655	0.336
**Anc 124**	0.564	0.42	0.237	0.245	1	0.619	0.66	0.481	0.484	0.649	0.631	0.645	0.649	0.229	0.186
**Node 10**	0.803	0.501	0.241	0.256	0.619	1	0.892	0.575	0.594	0.857	0.813	0.792	0.81	0.226	0.191
**Node 13**	0.746	0.503	0.254	0.268	0.66	0.892	1	0.584	0.606	0.953	0.897	0.858	0.876	0.232	0.205
**Node 253**	0.525	0.727	0.246	0.27	0.481	0.575	0.584	1	0.736	0.575	0.566	0.561	0.563	0.241	0.195
**Node 326**	0.533	0.583	0.224	0.256	0.484	0.594	0.606	0.736	1	0.612	0.592	0.584	0.588	0.225	0.19
**Node 384**	0.72	0.491	0.251	0.265	0.649	0.857	0.953	0.575	0.612	1	0.941	0.829	0.836	0.23	0.202
**Node 385**	0.689	0.472	0.248	0.26	0.631	0.813	0.897	0.566	0.592	0.941	1	0.795	0.798	0.232	0.197
**Node 3**	0.7	0.496	0.239	0.273	0.645	0.792	0.858	0.561	0.584	0.829	0.795	1	0.948	0.237	0.21
**Node 5**	0.72	0.5	0.241	0.264	0.649	0.81	0.876	0.563	0.588	0.836	0.798	0.948	1	0.229	0.206
***Pa*IPNS**	0.224	0.211	0.627	0.655	0.229	0.226	0.232	0.241	0.225	0.23	0.232	0.237	0.229	1	0.293
**Din11**	0.188	0.172	0.32	0.336	0.186	0.191	0.205	0.195	0.19	0.202	0.197	0.21	0.206	0.293	1

Figure S3. Sequence identity matrix for PK2 and Pd1 EFEs, reconstructed ancestors, *Pa*IPNS, and Din11. The colors denote the percentage of identity ranging from large (green) to small (red).

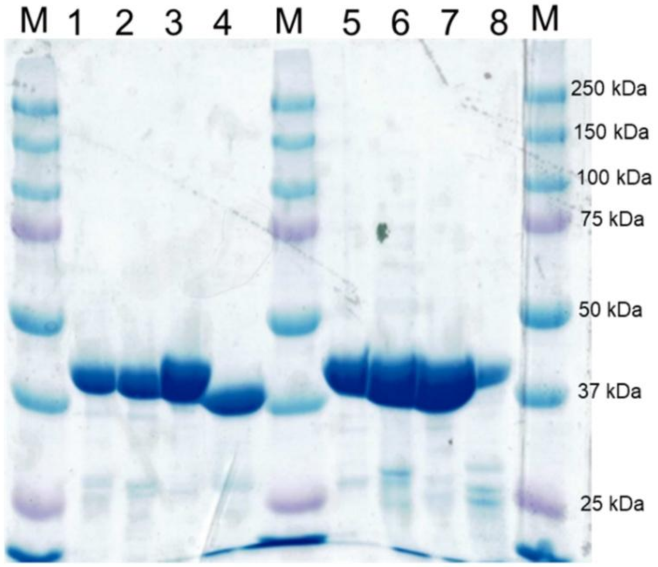

Figure S4. Purified ancestral proteins of EFE. The purified nodal ancestor proteins (3 to 7.5 μg) were analyzed by SDS-PAGE and compared to Precision Plus Protein Dual Color Standard markers (Bio-Rad) in lanes labeled M. The lanes containing proteins associated with each Node are (1) 3, (2) 5, (3) 10, (4) 13, (5) 253, (6) 326, (7) 384, and (8) 385.

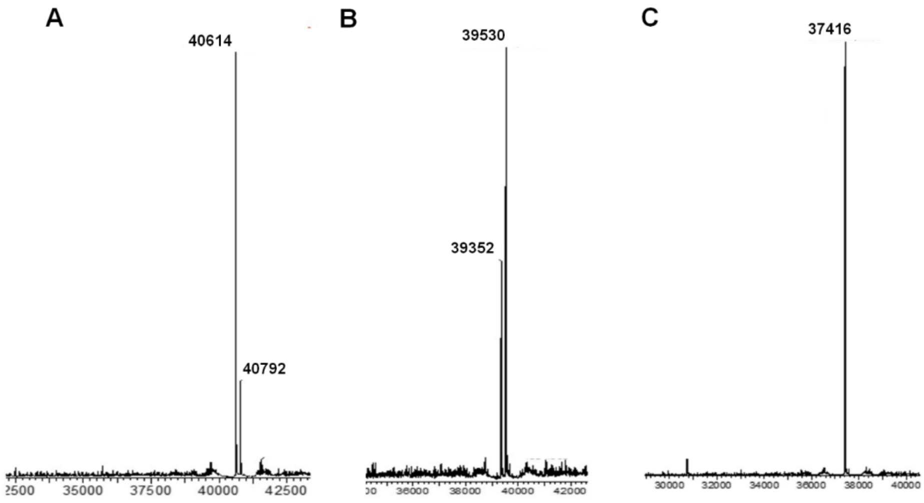

Figure S5. ESI-MS of Anc124 and Anc357. (A) His_6-tagged Anc124 (expected *m*/*z* 40,614). (B) His_6-tagged Anc357 (expected *m*/*z* 39,352). (C) TEV-cleaved Anc357 (expected *m*/*z* 37,413).

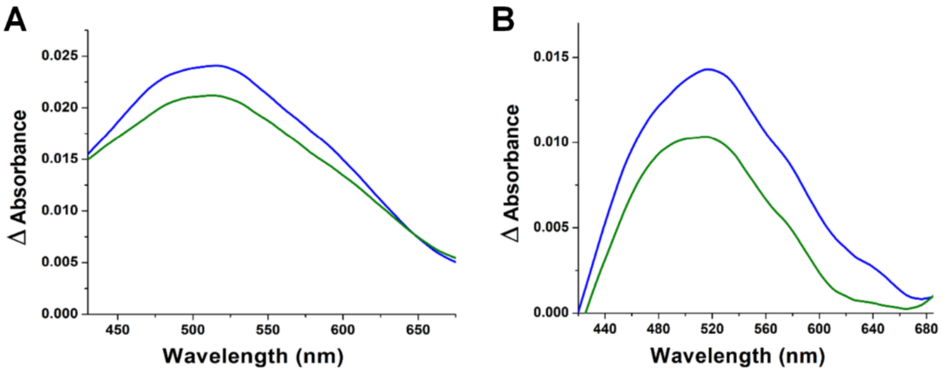

Figure S6. Difference absorbance spectra demonstrate the binding of 2OG to the Anc124 and Anc357 proteins. Difference spectra were generated using Fe(II)-bound (A) Anc124 protein (144 μM) and (B) Anc357 protein (141 μM) upon addition of 2OG (blue) and subsequent addition of L-Arg (green). The anaerobic samples containing 5.54 or 5.26 mg/mL of ancestor protein, respectively, 2 mM sodium dithionite, 1 mM $Fe(NH_4)_2(SO_4)_2$, 1 mM 2OG, and (when present) 1 mM L-Arg in 25 mM HEPES, pH 7.5.

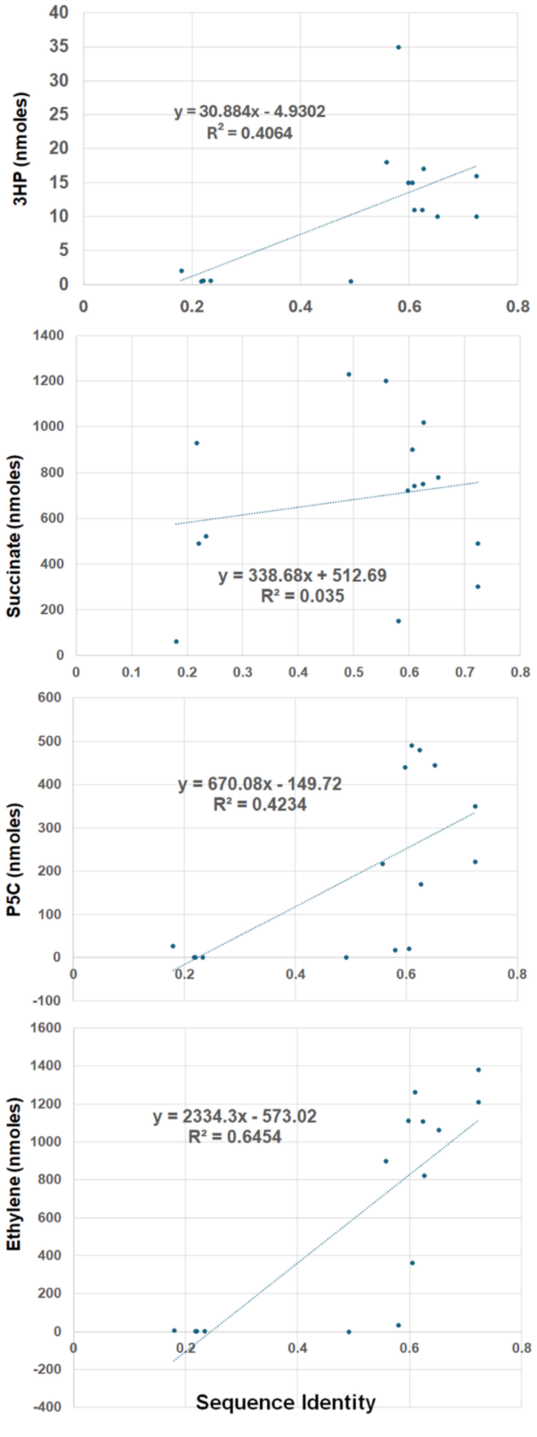

Figure S7. Correlations between the formation products (ethylene, P5C, succinate, and 3HP) and the sequence identities of ancestral proteins to the PK2 and Pd1 EFEs.

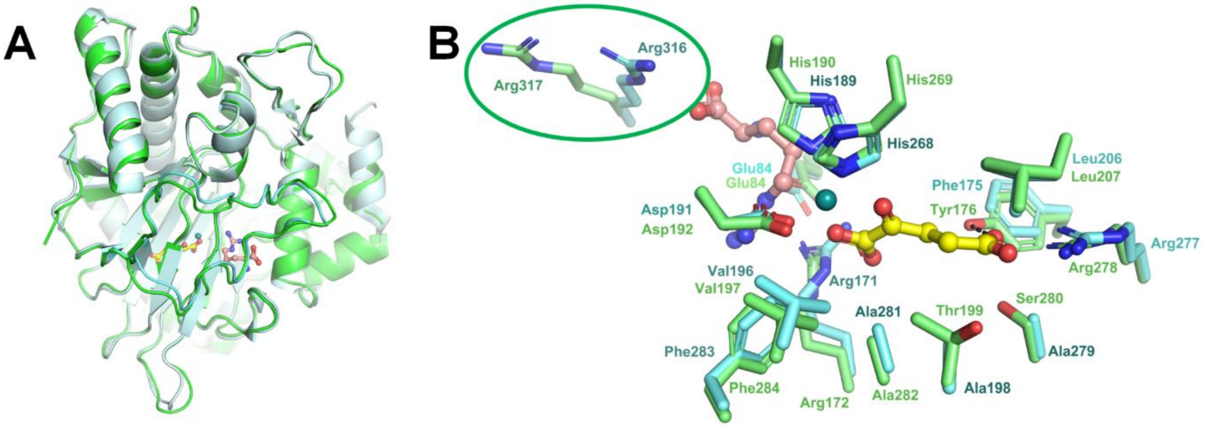

Figure S8. Superposition of the AlphaFold model of the Anc124 protein with PK2 EFE·Mn(II)·2OG·L-Arg (PDB: 5V2Y). (A) Cartoon depiction of the protein folds with PK2 EFE in cyan and the Anc124 protein in light green with selected components depicted as sticks. (B) Expanded view of the active sites for PK2 EFE and the Anc124 protein shown in stick view. Ball and stick views are shown for the PK2 EFE bound 2OG (yellow) and L-Arg (pink). Mn(II) is a cyan sphere. The distance between Anc124 Tyr176 and 2OG (dashed line) is 2.3 Å. The shift in position of Arg317/Arg316 (shown by the green oval) is attributed to the model's lack of L-Arg, which leads to a change in position of this residue in PK2 EFE.

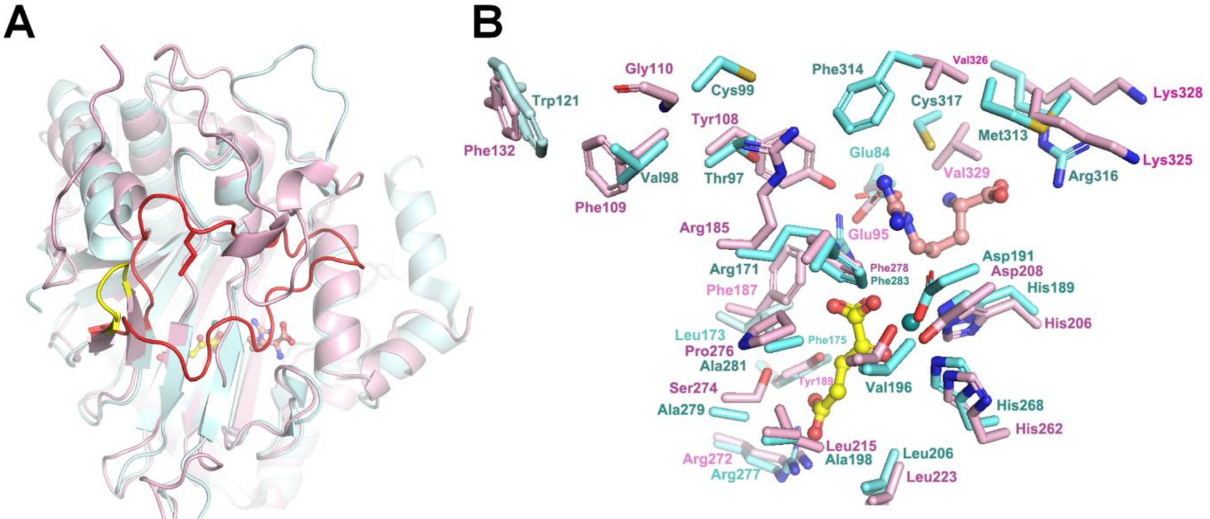

Figure S9. Superposition of the AlphaFold model of the Anc317 protein (pink) with PK2 EFE·Mn(II)·2OG·L-Arg (cyan) (PDB: 5V2Y). (A) Cartoon depiction of the protein folds emphasizing the long loop of PK2 EFE (red, containing Glu215 shown in stick view) that is replaced by a short loop in the Anc317 protein (yellow). (B) Expanded view of the active sites. Ball and stick views are shown for the PK2 EFE bound 2OG (yellow) and L-Arg (pink), and Mn(II) is a cyan sphere.

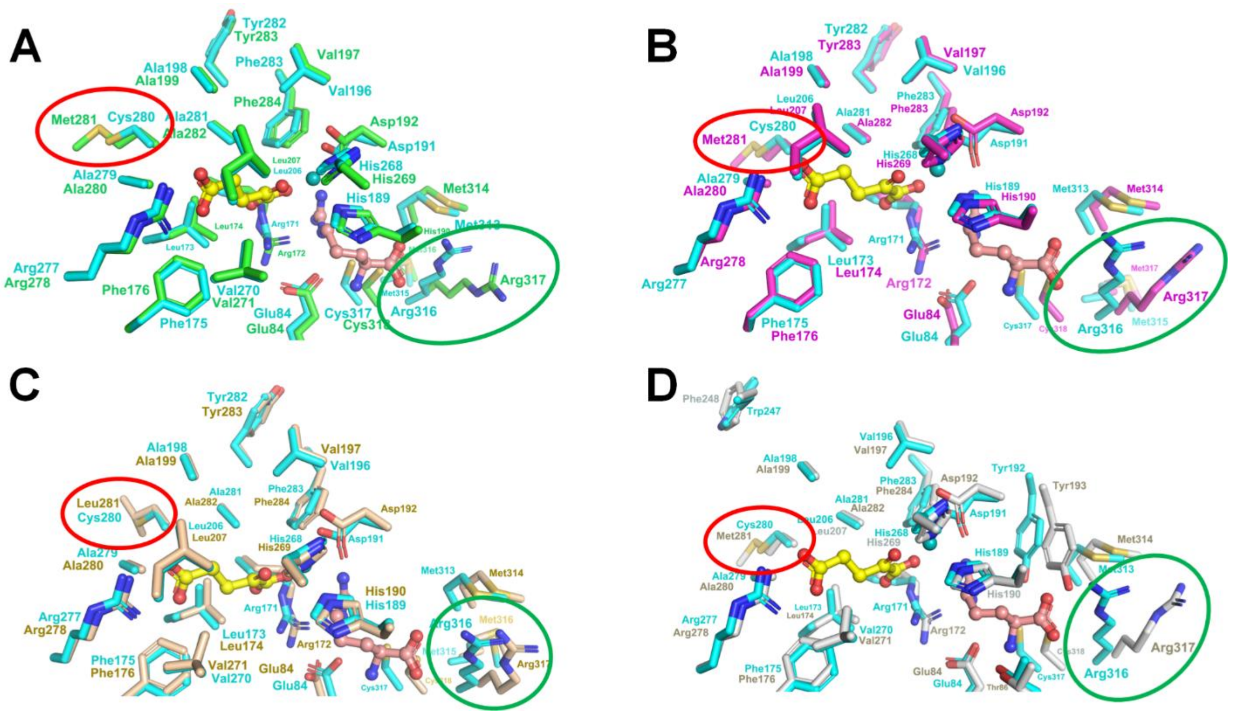

Figure S10. The active site residues for AlphaFold models of the (A) Node 3 (green), (B) Node 5 (magenta), (C) Node 10 (brown), and (D) Node 13 proteins (gray) are superpositioned with the corresponding active site residues of PK2 EFE·Mn(II)·2OG·L-Arg (cyan) (PDB: 5V2Y). Ball and stick views of 2OG (yellow) and L-Arg (pink) from the PK2 EFE structure are shown along with a cyan sphere for Mn(II). Each nodal protein has one residue in the region shown (indicated by the red ovals) that differs from that found in PK2 EFE. Large shifts of another residue (green ovals, Arg317 in the models or Arg316 in PK2 EFE) are attributed to changes due to binding L-Arg.

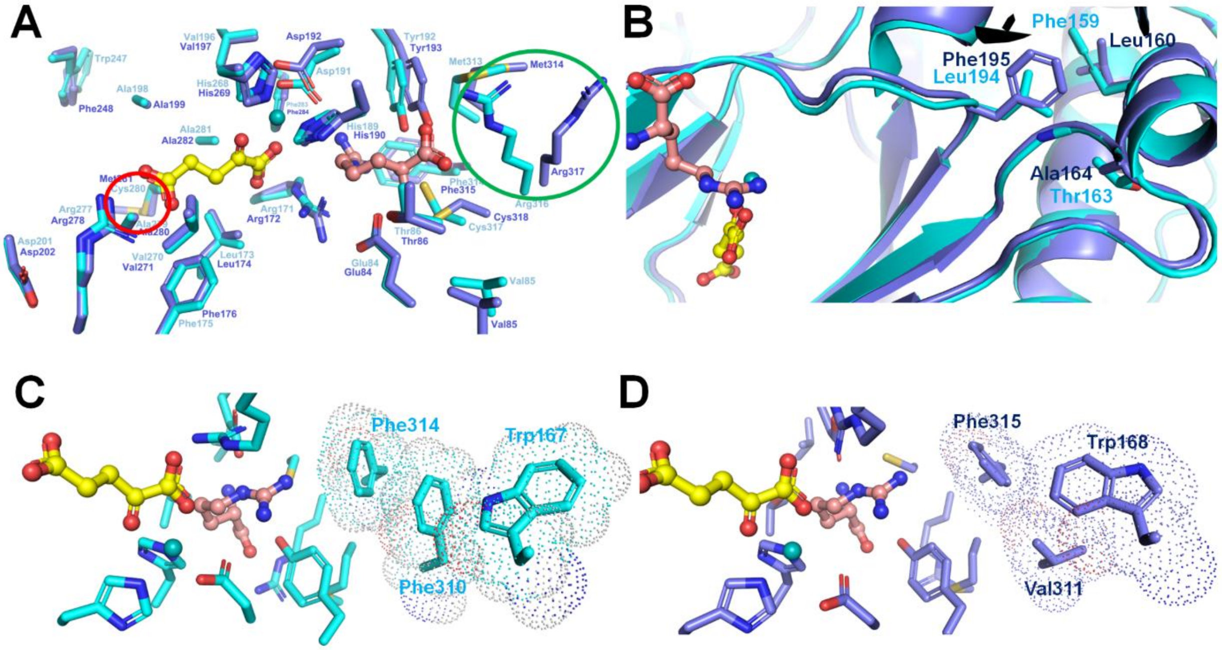

Figure S11. The active site residues for the AlphaFold model of the Node 384 protein (dark blue) versus the corresponding active site residues of PK2 EFE·Mn(II)·2OG·L-Arg (cyan) (PDB: 5V2Y). Ball and stick views of 2OG (yellow) and L-Arg (pink) from the PK2 EFE structure are shown along with a cyan sphere for Mn(II). (A) Superpositioned active site views of PK2 EFE and the protein associated with Node 384 highlighting Cys280 replaced by Met281 (red oval) and the differing positions of Arg316/Arg317 (green oval). (B) More distant changes in these proteins that might affect the mobility/stability of the active site pocket. Changes in the size and hydrophobicity of the L-Arg binding pocket are due to a change from (C) Phe310 in PK2 EFE to (D) Val311 in the Node 384 protein.

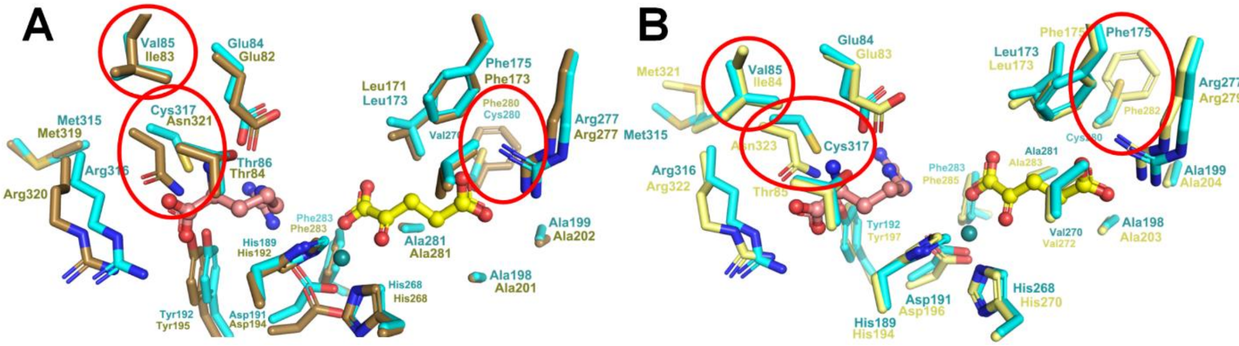

Figure S12. The active site residues for the AlphaFold models of the (A) Node 253 (brown) and (B) Node 326 (yellow) proteins versus the corresponding active site residues of PK2 EFE·Mn(II)·2OG·L-Arg (cyan) (PDB: 5V2Y). Ball and stick views of 2OG (yellow) and L-Arg (pink) from the PK2 EFE structure are shown along with a cyan sphere for Mn(II). Changes are indicated by the red ovals.

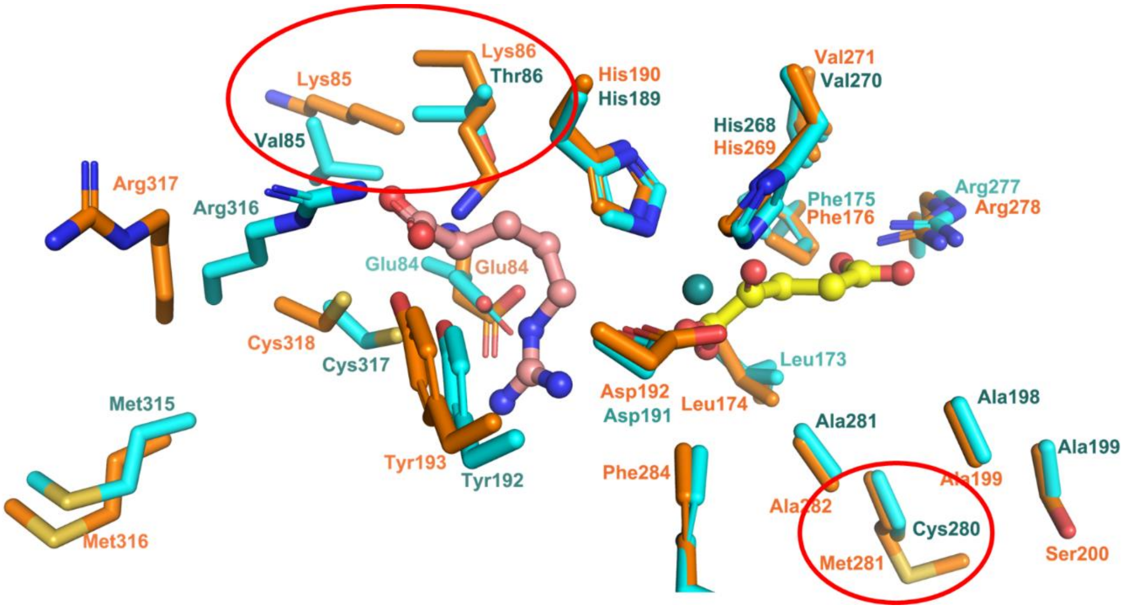

Figure S13. The active site residues for the AlphaFold model of the Node 385 protein (orange) versus the corresponding active site residues of PK2 EFE·Mn(II)·2OG·L-Arg (cyan) (PDB: 5V2Y). Ball and stick views of 2OG (yellow) and L-Arg (pink) from the PK2 EFE structure are shown along with a cyan sphere for Mn(II).

Table S1. Average Confidence Values for EFE Ancestral Nodes

Node	SH-aLRT[a]	UFB[b]	Average Residue Prediction Confidence
3	0	73	99.632
5	97.6	100	99.465
10	95.5	100	95.074
13	86.9	83	95.482
253	100	100	97.27
326	92.4	100	97.189
384	72.8	69	89.0
385	94.7	100	79.401

[a]SH-aLRT, Shimodaira-Hasegawa approximate likelihood ratio test. For node 3, the low SH-aLRT value is likely due to the difficulty in placing extant sequences WP_0302045871 and WP_2672449791 on the tree, causing poor confidence in the precise topology of the tree in this region

[b]UFB, ultrafast bootstrap approximation

Table S2. Primer Sequences and the Cloning Method Utilized

Construct	Primer sequences	Cloning method utilized
*Pa*IPNS	ATCCCATATGAGTACGCCATCCCTTCCAAT (Vector-F)<br>GGCTTTGTTAAAGAAGATCGCGACGTAATTGC (Vector-R)<br>ATGGCGTACTCATATGGGATTGGAAGTACAGGTTC (Insert-F)<br>CGATCTTCTTTAACAAAGCCCGAAAGGAAGC (Insert-R)	*In Vivo* Assembly (IVA)[1]
Anc124	GCATAGCATATGGCGGATTTTCAGAGCTTT (Insert-F)<br>CTACCTGGATCCCTACAGCACGC (Insert-R)	Inserted RE fragment into similarly digested plasmid
Anc317	GCATAGCATATGACCCAGGTGGTGAACG (Insert-F)<br>CTACCTGGATCCCAGCACGGTCTG (Insert-R)	Inserted RE fragment into similarly digested plasmid
Anc357	GCATAGCATATGACCAGCATGAGCGAACT (Insert-F)<br>CTACCTGGATCCCTATTCCAGCACTTCTTCG (Insert-R)	Inserted RE fragment into similarly digested plasmid
Node10	CCAATCCCATATGACTAACTTGCAGACGTTTGAACTGC (Insert-F)<br>GGCTTTGTTAGGAACGCTGCTTCATTTCTTCC (Insert-R)<br>GCAGCGTTCCTAACAAAGCCCGAAAGG (Vector-F)<br>GCAAGTTAGTCATATGGGATTGGAAGTACAGGTTC (Vector-R)	IVA
Node13	CCAATCCCATATGATGACCGATCTGCAAAC (Insert-F)<br>GGCTTTGTTATTGACGCAGTTCTTCTAACAC (Insert-R)<br>GAACTGCGTCAATAACAAAGCCCGAAAGG (Vector-F)	IVA

	GCAGATCGGTCATCATATGGGATTGGAAGTACAGG (Vector-R)	
Node253	CCAATCCCATATGTTGAAAACATTCACTTTGCC (Insert-F)<br>GGCTTTGTTAGTCTAACAGTTTCATTCTATC (Insert-R)<br>GAAACTGTTAGACTAACAAAGCCCGAAAGG (Vector-F)<br>GTGAATGTTTTCAACATATGGGATTGGAAGTACAGG (Vector-R)	IVA
Node326	GATGCTGGAA TAACAAAGCCCGAAAGGAAGC (Vector-F)<br>TCTGCAGATT CATATGGGATTGGAAGTACAGGTTC (Vector-R)	CloneExpress II One step Cloning kit from Vazyme
Node384	CAATCCCATATGATGACCAACCTTCAGACAC (Insert-F)<br>GCTTTGTTACTGTTTAAGTTCTTCTAAAACG (Insert-R)<br>GAACTTAAACAGTAACAAAGCCCGAAAGG (Vector-F)<br>GAAGGTTGGTCATCATATGGGATTGGAAGTACG (Vector-R)	IVA
Node385	GTACTTCCAATCCCA ATGATGACCAACCTGCA AACTTTTCACTTGC (Insert-F)<br>GCTTCCTTTCGGGCTTTGTTACTGCTTCAGCTCCTCTAAAACAC (Insert-R)<br>GAGGAGCTGAAGCAGTAACAAAGCCCGAAAGGAAGC (Vector-F)<br>GCAGGTTGGTCATCATATGGGATTGGAAGTACAGGTTC (Vector-R)	IVA
Node3	ATCCCATATGATGACAGATCTTCAGACATTCCACTT GCC (Insert-F)<br>GATCCTTAGCGCCTGCAAGCGCTGCCTC (Insert-R)<br>GCTTGCAGGCGCTAAGGATCCGAATTCG (Vector-F)<br>GATCTGTCATCATATGGGATTGGAAGTAC (Vector-R)	NEBuilder HiFi DNA Assembly Cloning Kit
Node5	ATCCCATATGATGACAGATTTGCAAACGTTC (Insert-F)<br>GATCCTTAGCAGCCCGTCTAAGAGCTTC (Insert-R)<br>TAGACGGGCTGCTAAGGATCCGAATTCG (Vector-F)<br>AATCTGTCATCATATGGGATTGGAAGTAC (Vector-R)	NEBuilder HiFi DNA Assembly Cloning Kit

Table S3. Data Collection and Refinement Statistics for Anc357 EFE·Mn(II)

Data Collection	
Beamline	LS-CAT 21-ID-D
Wavelength (Å)	1.127
Space group	P 1 21 1
Unit cell a, b, c (Å)<br>α, β, γ (°)	39.7 87.6 85.7<br>90.0 95.4 90.0
[a]Resolution (Å)	87.65 – 2.16 (2.23-2.16)
Unique reflections	31511 (2758)
[a]Redundancy	3.7 (3.6)
[a]Completeness (%)	100.0 (100.0)
[a] $I/\sigma I$	4.9 (1.2)
[a,b]R_{merge}	0.129 (0.893)
[c]$CC_{1/2}$	0.993 (0.572)
Refinement	
Protein atoms	4485
Ligand molecules	-
Metal	2 Mn
Water	250
[d]R_{work}/R_{free}	0.19/0.22
B-factors (Å^2)	36.9
Protein	37.2
Ligand	-
Metal	51.2
H_2O	33.4
R.m.s. deviation in bond lengths (Å)	0.008
R.m.s. deviation in bond angles (°)	1.02
Ramachandran plot (%) favored	99.8
Ramachandran plot (%) outliers	0.18
Rotamer outliers (%)	2.1
PDB ID	9OVH

[a]The highest resolution shell is shown in parentheses.
[b]$R_{merge} = \sum_{hkl} \sum_j |I_j(hkl) - \langle I(hkl) \rangle| / \sum_{hkl} \sum_j Ij(hkl)$, where I is the intensity of reflection.
[c]$CC_{1/2}$ is the correlation coefficient of the half datasets
[e]$R_{work} = \sum_{hkl} | |F_{obs}| - |F_{calc}| | / \sum_{hkl} |F_{obs}|$, where F_{obs} and F_{calc} is the observed and the calculated structure factor, respectively. R_{free} is the cross-validation R factor for the test set of reflections (5% of the total) omitted in model refinement.

[1] Garcia-Nafria, J., Watson, J. F., and Greger, I. H. (2016) IVA cloning: A single-tube universal cloning system exploiting bacterial *in vivo* assembly, *Sci. Rep. 6*, 27459.
